# Classification of early age facial growth pattern and identification of the genetic basis in two Korean populations

**DOI:** 10.1038/s41598-022-18127-6

**Published:** 2022-08-15

**Authors:** Mi-Yeon Cha, Yu-Jin Hong, Ja-Eun Choi, Tae-Song Kwon, Ig-Jae Kim, Kyung-Won Hong

**Affiliations:** 1 Theragen Bio Co., Ltd., 240 Pangyoyeok-ro, Seongnam-si, Gyeonggi-do 13493 Republic of Korea; 2 Center for Imaging Media Research, Korea Institute of Science and Technology, Seoul, 02792 Republic of Korea; 3 Human ICT CO., Ltd., 111, Dogok-ro, Gangnam-gu, Seoul, 06253 Republic of Korea

**Keywords:** Developmental biology, Genetics

## Abstract

Childhood to adolescence is an accelerated growth period, and genetic features can influence differences of individual growth patterns. In this study, we examined the genetic basis of early age facial growth (EAFG) patterns. Facial shape phenotypes were defined using facial landmark distances, identifying five growth patterns: continued-decrease, decrease-to-increase, constant, increase-to-decrease, and continued-increase. We conducted genome-wide association studies (GWAS) for 10 horizontal and 11 vertical phenotypes. The most significant association for horizontal phenotypes was rs610831 (TRIM29; β = 0.92, p-value = 1.9 × 10^−9^) and for vertical phenotypes was rs6898746 (ZSWIM6; β = 0.1103, p-value = 2.5 × 10^−8^). It is highly correlated with genes already reported for facial growth. This study is the first to classify and characterize facial growth patterns and related genetic polymorphisms.

## Introduction

Differences in the relative size, shape, and spatial arrangement (vertical, horizontal, and depth)^1^ of various facial features (e.g., eyes, nose, and lips) make each individual human face unique^2^. Therefore, skull growth and facial morphology are of interest^3^ to many scientific disciplines, especially anthropology, genetics, and forensic science^4^. Our face shapes change continuously from infanthood to adulthood. During the early ages, from 1 to 20 years, our face shapes grow rapidly, and genetic features may be responsible for individual differences in facial phenotypes. The period from childhood to adolescence is characterized by accelerated growth, and developmental modeling of facial morphology is useful for forensic and biomedical practices. Because the number of missing persons is increasing every year and technology is required to estimate the present face from the past. To understand early age facial growth, an important point is considering of the growth direction^5–7^.

The past 10 years of facial morphology research has benefited from the development of image recognition technology, which can quickly and accurately capture the details of the face^5^. Similarly, the development of genotyping technology facilitates the exploration of genetic impacts on human facial morphology phenotypes^3,6^. Although various studies have been conducted to examine facial growth, most studies have focused on identifying the causes of craniofacial abnormalities^7^. In the study of the healthy individuals of Europeans ancestry, some genes such as *MAFB, PAX9* associated with craniofacial development or syndromes^8^. The genetic loci associated with facial phenotypes were reported genes such as PRDM16, PAX3^9,10^, and TP63^9^. Research to investigate the biological basis underlying the normal range of facial variability has only recently been conducted. Over the past few years, as facial recognition technology has improved, substantial progress has been made in the identification of loci related to facial traits in published genome-wide association studies (GWAS)^11^. The starting point of GWAS analyses for facial morphology begins with craniofacial development or the identification of genetic loci associated with genetic facial deformities and syndromes. According to recently reported GWAS results^2^, studies on human phenotypes have identified and reported multiple loci associated with normal facial surface morphology.

While facial variation is influenced such as age and nutritional status, striking facial similarities within families reveal a strong genetic component^12–14^. However, genetic and GWAS studies are mainly studies of facial morphology in adults. The association between facial phenotype and SNP has been reported in European adolescents^15,16^, and facial changes (face height, eye width, and nose width) in 15-year-old British children have been studied^17^, but mostly adults. Therefore, understanding the relationship between facial shape and genetic loci during rapid facial changes period helps innate understanding of the face. It is expected to affect the overall industry necessary for children's awareness. This study was conducted on Korean subjects, the purpose is to increase the probability of identity inference by predicting the faces of long-term missing children. Therefore, it is important to understand the research in the period when the face shape changes rapidly.

A reported study of facial morphology in Koreans identified five GWAS loci associated with facial trats^18^ not for facial growth. In this study, we aimed to analyze early age facial growth (EAFG) patterns using facial landmark distances measured from normal facial surface phenotypes to examine the possible genetic basis of individual differences among two Korean populations.

## Results

### The overall population characteristics and measurements

**Figure 1:**
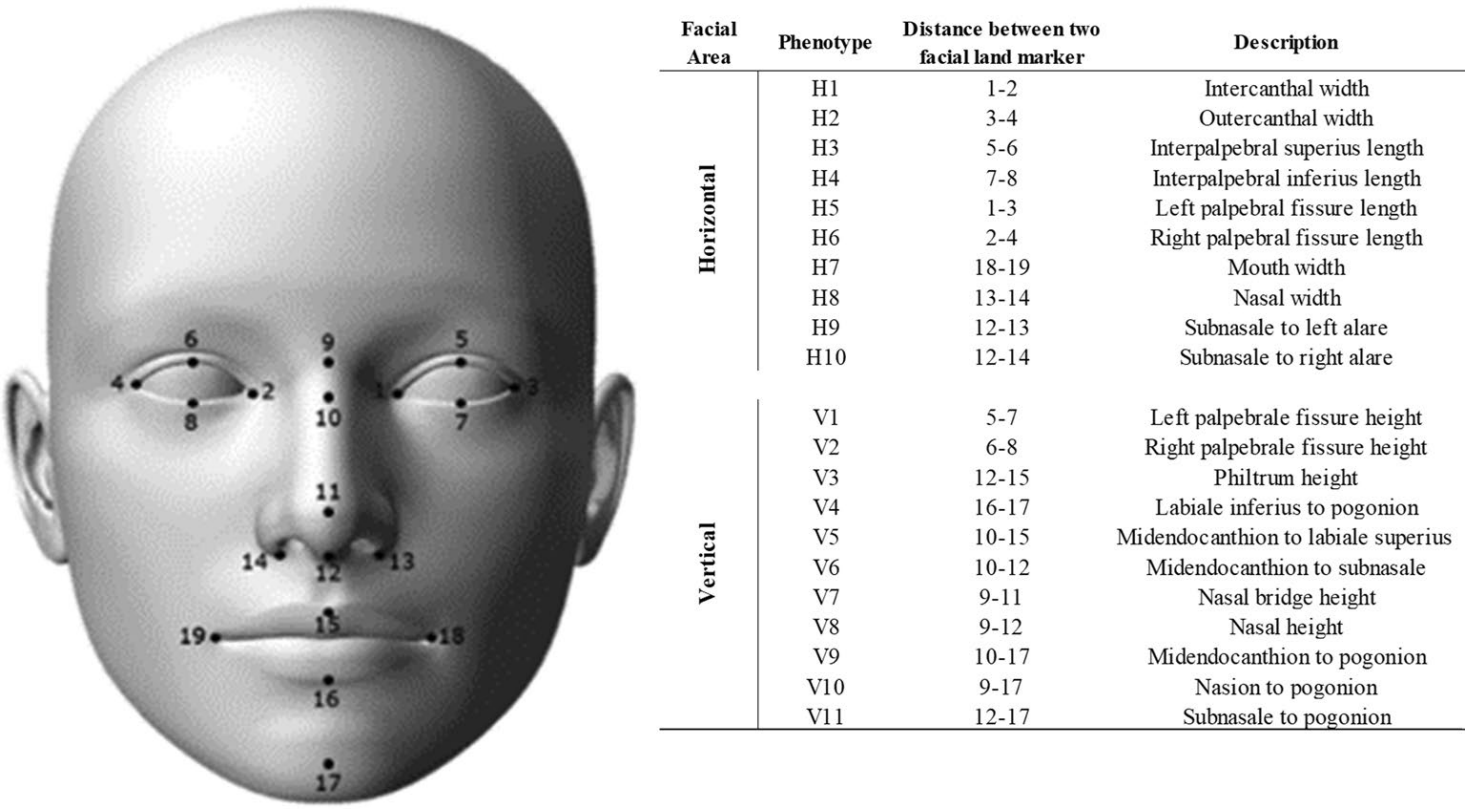
Measuring 19 facial landmarks using 21 facial measurements between facial landmark pairs.

We collected current and past photos from participants. For each participant, one current photo was obtained through studio photography, and 5 to 7 past frontal photos were collected. For the age of the past photos, 1 to 2 photos were collected and used for the study, each of which was less than 5 years old, 5 to 10 years old, 10 to 15 years old, and 15 to 20 years old. Supplementary Table S1 lists the number of photos collected by each participant and their age at the time of taking the photos in detail. The measurement data and characteristics information of each participant according to the facial area are shown in Fig. 1. Supplementary Table S2 shown the characteristics of the participant.

We quantified the facial features of two independent populations who were recruited during separate periods: 172 individuals in Population 1 (POP1) were recruited from January 2019 to July 2020, and 100 individuals in Population 2 (POP2) were recruited from July 2020 to September 2020. We collected a total of 172 current photos and 884 past photos for POP1 and 100 current photos and 600 past photos for POP2. The 21 facial phenotypes of each current and past profile photograph were determined by measuring the distances between 19 facial landmarks. Facial landmarks and measurement areas are depicted in Fig. 1, and the measurement results for each area are summarized in Table 1. All direct measurements were normalized against the distance between the center of left and right irises. The facial phenotypes were categorized into two facial groups: Category 1 was described as the horizontal index (H1–H10), and Category 2 was described as the vertical index (V1–V11).

**Figure 2:**
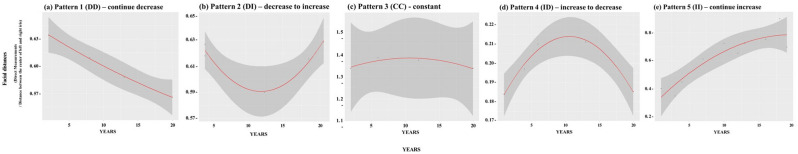
Definition of early age facial growth patterns.

**Table 1:**
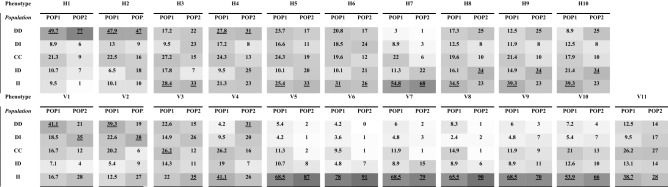
EAFG pattern distributions for each phenotype in two populations: populations 1 and 2.

The measurements from each past photograph were compared against those of the current photograph using non-linear regression methods to determine the facial changes over time. The regression patterns determined by the visual inspection of individual’s changes were used for the genetic association study (Supplementary Fig. S1).

### Defining each measurement and analyzing the time series of facial measurements according to age

The measurements from each past photograph were compared against those of the current photograph using non-linear regression methods to determine the facial changes over time. The facial growth patterns of each individual are determined by the visual inspection of individual’s changes were used for the genetic association study.

To understand the changes in facial measurements with age, a graph of the time series for each individual facial measurement was plotted for 21 phenotypes according to the age relative to the current age using a non-linear model. Supplementary Fig. S1A–C graphically represent individual growth patterns for each representative eye, nose, and mouth phenotype. Table 1 summarizes the distribution of facial growth patterns by face region in Pop1 and Pop2. We identified five EAFG patterns: Pattern 1 (DD), continued decrease; Pattern 2 (DI), decrease to increase; Pattern 3 (CC), constant; Pattern 4 (ID), increase to decrease; and Pattern 5 (II), continued increase (Fig. 2). The X-axis represents age, and the Y-axis represents facial distances between two selected points based on the 19 features measured. Among the EAFG patterns, the higher the frequency, the darker the gray. Among 21 phenotypes, 14 phenotypes, H1–H7 and V5–V11 showed similar distribution patterns between POP1 and POP2, whereas 7 phenotypes showed unique distribution patterns between POP1 and POP2. However, the 7 phenotypes representing unique growth patterns were clustered within Pattern 1 and Pattern 5. High proportions of Pattern 5 were observed for both horizontal and vertical measurements. When examining specific regions of the face, the area around the eyes showed high proportions of Pattern 1, whereas the other facial phenotypes showed high proportions of Pattern 5. For most Koreans, the distance between H1 and H2 decreased with aging, the measurement for H3 increased with age, and H4 showed a tendency to decrease with age. Among the other eye widths, H5 and H6, most commonly increased with age, and the vertical eye measurements, V1 and V2, also showed a tendency toward an increasing pattern. In the nose, the width of the nostrils most commonly increased, and the length of the vertical axis of the nose showed the largest increasing pattern. Around the lips, many individual’s patterns showed measurements that were maintained or increased types as they aging.

### Genotype analysis

Genome-wide single-nucleotide polymorphism (SNP) genotypes were obtained from an 800 K SNP microarray experiment using an Axiom array followed by imputation using 1000 Genomes Phase 3 data^18^, resulting in a total of 7,375,270 polymorphic SNPs included in the GWAS. We conducted a GWAS for the combined analysis of POP1 and POP2, in addition to separate analyses for POP1 and POP2. The significant or suggestive SNPs from the combined analysis were determined based on the criteria of a p-value < 5 × 10^−8^ for significant SNPs and 5 × 10^−8^ ≤ p-value < 1 × 10^−5^ for suggestive SNPs. In addition, SNPs in the individual POP1 and POP2 analysis with p-value < 0.05 were considered significant. The combined GWAS results are illustrated using quantile–quantile (QQ) plots (Supplementary Fig. S2) and Manhattan plots (Supplementary Fig. S3) for each phenotype. A total of 97 SNPs satisfied the genome-wide significance criteria (p-values < 5 × 10^−8^), and 759 SNPs were identified with suggestive association p-values (5 × 10^−8^ ≤ p-value < 1 × 10^−5^) for 21 facial phenotypes (Supplementary Table S3). The SNPs were analyzed by clustering patterns: 77 SNPs were singletons (i.e., a single significant SNP without co-segregation with other SNPs nearby the significant SNP), and 729 SNPs in 104 loci showed a clustered pattern (i.e., significant or suggestive SNPs co-segregated with more than three other significant or suggestive SNPs). The significant or suggestive and clustered SNP loci were illustrated using regional signal plots (Supplementary Fig. S4). The top significant SNPs for each significant cluster are described in Tables 2 and 3. The criteria for dividing DD, DI, CC, ID, and II patterns were established and validated using quantitative trait association analysis (Wald test). Among the 5 EAFG patterns in the horizontal and the vertical measurements, we identified significant and suggestive SNPs. The phenotype used in this study was analyzed by coding facial growth patterns from the past to the present from 1 to 5 (Supplementary Table S4). There was no significant difference in the distribution of patterns between males and females in the facial growth pattern. Therefore, gender was not used as a covariate in the GWAS analysis in this study.

**Table 2:** Features of suggestive and significant SNPs in horizontal regions associated with the 5 EAFG patterns identified in the combined sample and each individual population sample.

Phenotype	CHR:POS^*^	SNP	Alleles (ALT/REF)	This study	EAS	EUR	AMR	Combined population 1 & 2			Population 1			Population 2			Gene annotation	Gene description
								Beta	P	Bonferroni	Beta	P	Bonferroni	Beta	P	Bonferroni		
**H1**	**chr7: 4759542**	**rs4523152**	**A/G**	**0.12**	**0.13**	**0.36**	**0.22**	**0.75**	**2.2.E−06**	1.8.E+00	**0.75**	**2.0.E−04**	1.6.E+02	**0.57**	**1.1.E−02**	9.1.E+03	**FOXK1**	**Forkhead box K1**
**H1**	**chr11: 119897453**	**rs610831**	**C/ A**	**0.15**	**0.16**	**0.36**	**0.36**	**0.92**	**1.9.E−09**	1.5.E−03	**0.97**	**9.8.E−07**	7.9.E−01	**0.63**	**2.5.E−03**	2.0.E+03	**TRIM29**	**Tripartite motif containing 29**
H1	chr15: 25158591	rs75095637	C/A	0.3	0.33	0.08	0.08	− 0.54	9.5.E−06	7.6.E+00	− 0.67	1.4.E−04	1.1.E+02	− 0.34	1.4.E−02	1.2.E+04	SNRPN	Small nuclear ribonucleoprotein polypeptide N
H1	chr18: 5957480	rs609014	T/C	0.13	0.07	0.05	0.17	0.77	9.3.E−06	7.4.E+00	0.81	2.7.E−04	2.2.E+02	0.53	3.0.E−02	2.4.E+04	L3MBTL4	L3MBTL histone methyl-lysine binding protein 4
**H2**	**chr2: 61987760**	**rs12464486**	**A/G**	**0.17**	**0.12**	**0.2**	**0.2**	**0.82**	**6.1.E−07**	4.9.E−01	**1**	**9.9.E−07**	7.9.E−01	**0.55**	**4.8.E−02**	3.8.E+04	**FAM161A**	**FAM161 centrosomal protein A**
**H2**	**chr6: 98785243**	**rs1078326**	**A/G**	**0.41**	**0.41**	**0.49**	**0.43**	**− 0.56**	**5.0.E−06**	4.0.E+00	**− 0.63**	**3.7.E−05**	3.0.E+01	**− 0.45**	**3.3.E−02**	2.6.E+04	**POU3F2**	**POU class 3 homeobox 2**
H2	chr10: 115326193	rs3850690	G/A	0.43	0.39	0.04	0.37	0.57	7.4.E−06	5.9.E+00	0.62	5.5.E−05	4.4.E+01	0.49	3.2.E−02	2.6.E+04	HABP2	Hyaluronan binding protein 2
H2	chr20: 13573032	rs73088095	A/G	0.16	0.18	0.16	0.11	0.7	1.9.E−06	1.5.E+00	0.62	5.9.E−04	4.7.E+02	0.89	6.1.E−04	4.9.E+02	TASP1	Taspase 1
H3	chr1: 199634598	rs10919736	A/G	0.39	0.4	0.27	0.31	0.57	7.6.E−06	6.1.E+00	0.47	2.0.E−03	1.6.E+03	0.75	8.2.E−04	6.6.E+02	NR5A2	Nuclear receptor subfamily 5 group a member 2
**H3**	**chr7: 95531815**	**rs4357216**	**T/C**	**0.54**	**0.47**	**0.87**	**0.86**	**− 0.57**	**9.0.E−06**	7.2.E+00	**− 0.53**	**1.0.E−03**	8.2.E+02	**− 0.64**	**3.1.E−03**	2.5.E+03	**DYNC1I1**	**Dynein cytoplasmic 1 intermediate chain 1**
H3	chr9: 90275422	rs59370521	G/ A	0.37	0.47	0.44	0.28	− 0.65	1.1.E−06	8.5.E−01	− 0.63	9.9.E−05	7.9.E+01	− 0.68	4.3.E−03	3.4.E+03	DAPK1	Death associated protein kinase 1
H3	chr10: 86454472	rs11201173	T/C	0.4	0.44	0.29	0.17	0.58	3.9.E−06	3.1.E+00	0.57	1.6.E−04	1.3.E+02	0.58	1.1.E−02	8.7.E+03	CCSER2	Coiled-coil serine rich protein 2
H3	chr10: 86052147	rs10788337	G/A	0.75	0.76	0.61	0.55	− 0.68	5.3.E−06	4.2.E+00	− 0.6	1.8.E−03	1.4.E+03	− 0.74	2.0.E−03	1.6.E+03	LINC00858	Long intergenic non-protein coding RNA 858
**H3**	**chr12: 132189183**	**rs11246765**	**T/C**	**0.5**	**0.54**	**0.14**	**0.22**	**− 0.59**	**7.7.E−06**	6.1.E+00	**− 0.51**	**3.0.E−03**	2.4.E+03	**− 0.7**	**7.4.E−04**	6.0.E+02	**SFSWAP**	**Splicing factor SWAP**
H4	chr6: 28178636	rs16893817	G/A	0.21	0.16	0.16	0.18	0.67	7.6.E−06	6.1.E+00	0.62	8.8.E−04	7.0.E+02	0.75	3.8.E−03	3.0.E+03	TOB2P1	Transducer of ERBB2, 2 pseudogene 1
H4	chr7: 95531815	rs4357216	T/C	0.54	0.47	0.87	0.86	0.6	5.8.E−06	4.6.E+00	0.45	8.5.E−03	6.8.E+03	0.83	1.1.E−04	8.4.E+01	DYNC1I1	Dynein cytoplasmic 1 intermediate chain 1
H4	chr8: 53365524	rs188209	C/G	0.75	0.77	0.73	0.72	0.68	6.1.E−06	4.9.E+00	0.71	3.8.E−04	3.0.E+02	0.62	8.6.E−03	6.9.E+03	ST18	ST18 C2H2C-type zinc finger transcription factor
H4	chr10: 86447497	rs59213736	T/G	0.39	0.44	0.29	0.19	− 0.59	5.8.E−06	4.6.E+00	− 0.59	2.2.E−04	1.7.E+02	− 0.57	1.0.E−02	8.2.E+03	CCSER2	Coiled-coil serine rich protein 2
H4	chr10: 37425238	rs1200907	G/A	0.28	0.33	0.59	0.48	0.6	9.3.E−06	7.4.E+00	0.55	9.4.E−04	7.5.E+02	0.67	3.3.E−03	2.6.E+03	ANKRD30A	Ankyrin repeat domain 30A
H5	chr12: 70403306	rs1239925	T/C	0.36	0.34	0.69	0.74	− 0.68	1.4.E−06	1.1.E+00	− 0.68	1.2.E−04	9.4.E+01	− 0.61	8.0.E−03	6.4.E+03	MYRFL	Myelin regulatory factor like
H5	chr16: 10268243	rs1104474	C/ T	0.47	0.46	0.67	0.58	0.56	9.2.E−06	7.3.E+00	0.39	1.7.E−02	1.4.E+04	0.79	4.0.E−05	3.2.E+01	GRIN2A	Glutamate ionotropic receptor NMDA type subunit 2A
H6	chr1: 240747943	rs4571950	G/A	0.36	0.46	0.21	0.27	− 0.63	4.3.E−06	3.4.E+00	− 0.65	1.3.E−04	1.0.E+02	− 0.57	1.2.E−02	9.6.E+03	GREM2	Gremlin 2, DAN family BMP antagonist
H6	chr1: 92098152	rs12137034	A/ G	0.44	0.49	0.42	0.37	− 0.62	5.6.E−06	4.5.E+00	− 0.68	9.8.E−05	7.8.E+01	− 0.52	1.9.E−02	1.5.E+04	TGFBR3	Transforming growth factor beta receptor 3
H6	chr8: 98302185	rs2635140	G/A	0.11	0.16	0.34	0.31	0.89	2.8.E−06	2.2.E+00	0.94	6.8.E−05	5.4.E+01	0.78	1.5.E−02	1.2.E+04	LOC101927066	Uncharacterized LOC101927066
H6	chr9: 31411344	rs10813575	T/ G	0.36	0.27	0.19	0.32	0.61	6.7.E−06	5.4.E+00	0.68	9.2.E−05	7.4.E+01	0.5	2.3.E−02	1.9.E+04	ACO1	Aconitase 1
H6	chr11: 125839926	rs562363	C/T	0.23	0.22	0.17	0.24	0.73	1.2.E−06	9.6.E−01	0.85	2.5.E−05	2.0.E+01	0.58	1.2.E−02	9.6.E+03	CDON	Cell adhesion associated, oncogene regulated
H6	chr14: 81237260	rs1976104	C/ T	0.22	0.13	0.15	0.22	− 0.69	5.3.E−06	4.2.E+00	− 0.72	1.6.E−04	1.3.E+02	− 0.62	1.2.E−02	9.6.E+03	CEP128	Centrosomal protein 128
**H7**	**chr3: 151243183**	**rs62274696**	**C/G**	**0.13**	**0.14**	**0.01**	**0.02**	**− 0.81**	**2.1.E−08**	1.7.E−02	**− 0.82**	**2.1.E−05**	1.7.E+01	**− 0.7**	**2.1.E−04**	1.6.E+02	**IGSF10**	**Immunoglobulin superfamily member 10**
H7	chr4: 9809859	rs12500086	G/A	0.36	0.4	0.19	0.33	− 0.43	5.1.E−06	4.1.E+00	− 0.43	6.3.E−04	5.0.E+02	− 0.33	8.2.E−03	6.6.E+03	SLC2A9	Solute carrier family 2 member 9
H7	chr11: 112696012	rs7115108	T/C	0.2	0.24	0.28	0.19	− 0.67	7.3.E−09	5.8.E−03	− 0.77	3.6.E−07	2.9.E−01	− 0.35	3.5.E−02	2.8.E+04	LOC101928847	Uncharacterized LOC101928847
H7	chr13: 62363197	rs9539309	G/A	0.17	0.21	0.27	0.23	− 0.57	2.9.E−06	2.3.E+00	− 0.59	2.6.E−04	2.1.E+02	− 0.45	5.2.E−03	4.2.E+03	LINC00358	Long intergenic non-protein coding RNA 358
H8	chr7: 103948904	rs4729991	G/ A	0.19	0.23	0.04	0.29	− 0.8	8.3.E−06	6.7.E+00	− 0.82	2.3.E−04	1.9.E+02	− 0.76	1.3.E−02	1.0.E+04	LHFPL3	LHFPL tetraspan subfamily member 3
H9	chr2: 105819699	rs57392464	G/A	0.15	0.2	0	0.01	− 0.74	7.5.E−06	6.0.E+00	− 0.61	4.1.E−03	3.3.E+03	− 0.91	5.1.E−04	4.1.E+02	GPR45	G protein-coupled receptor 45
**H9**	**chr4: 30164906**	**rs79854133**	**C/T**	**0.14**	**0.08**	**0.01**	**0.05**	**− 0.94**	**2.3.E−06**	1.9.E+00	**− 1.01**	**3.2.E−05**	2.6.E+01	**− 0.83**	**1.7.E−02**	1.3.E+04	**PCDH7**	**Protocadherin 7**
H9	chr7: 82791402	rs4341113	C/A	0.13	0.13	0.65	0.5	− 0.81	8.7.E−06	7.0.E+00	− 0.86	4.3.E−04	3.4.E+02	− 0.71	1.1.E−02	8.4.E+03	PCLO	Piccolo presynaptic cytomatrix protein
H9	chr17: 72633223	rs8069701	A/G	0.34	0.28	0.42	0.3	− 0.6	7.5.E−06	6.0.E+00	− 0.58	3.9.E−04	3.1.E+02	− 0.64	4.8.E−03	3.9.E+03	CD300E	CD300 molecule like family member E
H9	chr22: 32737548	rs5753988	T/A	0.35	0.34	0.04	0.09	0.63	5.3.E−07	4.3.E−01	0.65	1.8.E−05	1.5.E+01	0.56	1.3.E−02	1.0.E+04	SLC5A4-RFPL3	Solute carrier family 5 member 4
H9	chr22: 32823156	rs5749436	A/G	0.44	0.45	0.14	0.17	0.57	6.4.E−06	5.1.E+00	0.53	4.7.E−04	3.8.E+02	0.62	6.4.E−03	5.1.E+03	BPIFC	BPI fold containing family C
**H10**	**chr2:137331155**	**rs2060004**	**T/A**	**0.13**	**0.13**	**0.26**	**0.21**	**− 0.75**	**3.1.E−06**	2.5.E+00	**− 0.63**	**2.3.E−03**	1.8.E+03	**− 0.81**	**1.7.E−03**	1.3.E+03	**CXCR4**	**C-X-C motif chemokine receptor 4**
H10	chr3:175898398	rs12488870	A/T	0.36	0.35	0.27	0.25	0.61	2.8.E−06	2.3.E+00	0.65	2.5.E−05	2.0.E+01	0.55	1.8.E−02	1.5.E+04	NAALADL2	N-acetylated alpha-linked acidic dipeptidase like 2
H10	chr4:131116188	rs1873867	T/C	0.29	0.25	0.2	0.26	− 0.65	3.5.E−06	2.8.E+00	− 0.55	1.7.E−03	1.4.E+03	− 0.68	3.1.E−03	2.5.E+03	LOC101927305	Uncharacterized LOC101927305
H10	chr5:35805553	rs1389836	C/T	0.1	0.09	0.26	0.16	− 0.79	7.3.E−06	5.8.E+00	− 0.77	3.4.E−04	2.7.E+02	− 0.74	1.2.E−02	9.6.E+03	SPEF2	Sperm flagellar 2
H10	chr7:55826399	rs144020759	C/G	0.19	0.16	0.02	0.04	0.72	4.0.E−06	3.2.E+00	0.54	2.2.E−03	1.7.E+03	1.02	6.4.E−04	5.1.E+02	14-Sep	Septin 14
**H10**	**chr9:134423332**	**rs9776117**	**C/G**	**0.32**	**0.3**	**0.27**	**0.3**	**− 0.6**	**5.2.E−06**	4.2.E+00	**− 0.49**	**1.4.E−03**	1.1.E+03	**− 0.77**	**1.4.E−03**	1.2.E+03	**RAPGEF1**	**Rap guanine nucleotide exchange factor 1**
H10	chr10:121108681	rs4751713	G/A	0.15	0.13	0.01	0.11	− 0.86	3.1.E−08	2.5.E−02	− 0.8	3.5.E−05	2.8.E+01	− 0.9	4.4.E−04	3.5.E+02	GRK5	G protein-coupled receptor kinase 5
H10	chr10:100013977	rs878178	T/A	0.25	0.22	0.32	0.2	− 0.63	6.1.E−06	4.8.E+00	− 0.47	3.4.E−03	2.7.E+03	− 0.98	1.5.E−04	1.2.E+02	LOXL4	Lysyl oxidase like 4
H10	chr13:21569905	rs56375211	A/T	0.16	0.15	0.34	0.25	− 0.73	3.2.E−06	2.6.E+00	− 0.76	7.7.E−05	6.1.E+01	− 0.59	2.7.E−02	2.1.E+04	LATS2	Large tumor suppressor kinase 2
H10	chr19:7874562	rs58009080	C/G	0.31	0.27	0.05	0.23	− 0.6	3.7.E−06	3.0.E+00	− 0.54	1.5.E−03	1.2.E+03	− 0.6	3.5.E−03	2.8.E+03	EVI5L	Ecotropic viral integration site 5 like

Significant values are in bold.

*Physical positions are based on NCBI build 37 of the human genome.

**Table 3:** Features of suggestive and significant SNPs in vertical regions associated with the 5 EAFG patterns identified in the combined sample and in each individual population.

Phenotype	CHR:POS^*^	SNP	Alleles (ALT/REF)	This study	EAS	EUR	AMR	Combined population 1 & 2			Population 1			Population 2			Gene annotation	Gene description
								Beta	P^†^	Bonferroni	Beta	P^††^	Bonferroni	Beta	P^†††^	Bonferroni		
V1	chr2: 147796276	rs1033211	T/C	0.12	0.11	0.06	0.06	− 1.02	3.4.E−06	2.8.E+00	− 0.97	4.9.E−04	3.9.E+02	− 1.09	2.2.E−03	1.7.E+03	ACVR2A	Activin A receptor type 2A
V1	chr13: 22791159	rs12585338	T/C	0.28	0.28	0.01	0.06	− 0.7	4.6.E−07	3.7.E−01	− 0.66	1.0.E−04	8.1.E+01	− 0.74	2.3.E−03	1.8.E+03	LINC00540	Long intergenic non-protein coding RNA 540
V1	chr14: 23711119	rs12880128	G/A	0.44	0.39	0.45	0.28	− 0.6	3.8.E−06	3.0.E+00	− 0.54	6.3.E−04	5.1.E+02	− 0.67	4.8.E−03	3.8.E+03	RNF212B	Ring finger protein 212B
V1	chr17: 76793533	rs3744803	T/C	0.14	0.11	0	0.01	0.87	5.8.E−06	4.6.E+00	0.92	1.1.E−04	8.9.E+01	0.81	1.2.E−02	9.2.E+03	USP36	Ubiquitin specific peptidase 36
V1	chr19: 32976941	rs35641247	T/G	0.33	0.31	0.06	0.08	0.64	2.2.E−06	1.7.E+00	0.55	1.2.E−03	9.5.E+02	0.71	1.3.E−03	1.0.E+03	DPY19L3	Dpy-19 like C-mannosyltransferase 3
**V1**	**chr21: 46777852**	**rs2838896**	**T/C**	**0.55**	**0.49**	**0.46**	**0.32**	**0.57**	**4.0.E**−**06**	3.2.E+00	**0.48**	**1.1.E**−**03**	8.8.E+02	**0.8**	**1.7.E**−**04**	1.3.E+02	**COL18A1**	**Collagen Type XVIII alpha 1 chain**
V2	chr1: 226982919	rs34020484	A/G	0.25	0.29	0.33	0.27	0.65	9.0.E−06	7.2.E+00	0.47	7.9.E−03	6.4.E+03	0.86	4.6.E−04	3.7.E+02	ITPKB	Inositol-trisphosphate 3-kinase B
V2	chr6: 35081931	rs2476822	A/G	0.58	0.51	0.5	0.54	0.56	3.9.E−06	3.1.E+00	0.45	2.5.E−03	2.0.E+03	0.69	4.7.E−04	3.8.E+02	TCP11	T-complex 11
V2	chr15: 39024657	rs8042858	T/C	0.11	0.09	0.25	0.16	0.89	4.0.E−06	3.2.E+00	0.92	1.2.E−04	9.4.E+01	0.79	1.2.E−02	9.3.E+03	C15orf54	Long intergenic non-protein coding RNA 2694
V2	chr17: 80628142	rs56137100	T/C	0.41	0.38	0.32	0.38	0.57	7.1.E−06	5.7.E+00	0.56	3.1.E−04	2.5.E+02	0.49	2.4.E−02	1.9.E+04	MIR4525	MicroRNA 4525
**V3**	**chr8: 15809615**	**rs11203737**	**G/C**	**0.77**	**0.77**	**0.93**	**0.74**	**0.69**	**5.2.E**−**06**	4.2.E+00	**0.49**	**9.4.E**−**03**	7.5.E+03	**0.99**	**1.0.E**−**04**	8.2.E+01	**TUSC3**	**Tumor Suppressor candidate 3**
**V3**	**chr18: 27282027**	**rs9748670**	**A/T**		**0.35**	**0.08**	**0.15**	**− 0.66**	**5.2.E**−**07**	4.2.E−01	**− 0.6**	**1.3.E**−**04**	1.0.E+02	**− 0.76**	**1.5.E**−**03**	1.2.E+03	**CHST9**	**Carbohydrate Sulfotransferase 9**
V4	chr3: 105962893	rs1020365	C/T	0.12	0.08	0.01	0.08	− 0.77	8.2.E−06	6.6.E+00	− 0.78	1.6.E−03	1.3.E+03	− 0.79	1.4.E−03	1.1.E+03	CBLB	Metabolism of cobalamin associated B
V4	chr9: 110431889	rs62569569	A/G	0.3	0.36	0.14	0.24	− 0.49	8.8.E−06	7.0.E+00	− 0.45	1.1.E−03	9.0.E+02	− 0.57	2.5.E−03	2.0.E+03	ACTL7B	Actin like 7B
V4	chr12: 47427972	rs201159408	A/G	0.17	0.14	0.19	0.21	− 0.63	8.5.E−06	6.8.E+00	− 0.61	2.1.E−04	1.7.E+02	− 0.69	1.3.E−02	1.1.E+04	LINC02156	Long intergenic non-protein coding RNA 2156
V5	chr2: 101596624	rs3768988	G/A	0.32	0.36	0.09	0.32	− 0.47	8.8.E−07	7.0.E−01	− 0.53	7.4.E−05	5.9.E+01	− 0.33	2.8.E−03	2.2.E+03	LOC101927142	Uncharacterized LOC101927142
V5	chr2: 101579979	rs74509671	A/G	0.39	0.39	0.1	0.32	− 0.43	3.1.E−06	2.4.E+00	− 0.48	2.1.E−04	1.7.E+02	− 0.31	2.5.E−03	2.0.E+03	NPAS2	Neuronal PAS domain protein 2
V5	chr4: 71122281	rs13146558	G/T	0.24	0.22	0.37	0.41	− 0.48	3.9.E−06	3.1.E+00	− 0.45	1.6.E−03	1.3.E+03	− 0.44	6.1.E−04	4.9.E+02	CABS1	Calcium binding protein, spermatid associated 1
**V5**	**chr5: 60843706**	**rs6898746**	**A/T**	**0.15**	**0.21**	**0.52**	**0.55**	**− 0.7**	**2.5.E**−**08**	2.0.E−02	**− 0.82**	**2.3.E**−**06**	1.8.E+00	**− 0.45**	**2.5.E**−**03**	2.0.E+03	**ZSWIM6**	**Zinc Finger SWIM-type containing 6**
V5	chr10: 121226365	rs11198944	T/C	0.12	0.08	0.18	0.15	− 0.61	6.6.E−06	5.2.E+00	− 0.7	5.7.E−04	4.6.E+02	− 0.47	7.7.E−04	6.1.E+02	GRK5	G protein-coupled receptor kinase 5
V5	chr11: 98482398	rs77519679	A/G	0.19	0.2	0.32	0.27	− 0.55	1.4.E−07	1.1.E−01	− 0.63	3.2.E−05	2.6.E+01	− 0.42	1.6.E−04	1.3.E+02	CNTN5	Contactin 5
V6	chr2: 235806038	rs9789458	T/C	0.25	0.26	0.21	0.3	− 0.39	9.0.E−06	7.2.E+00	− 0.51	1.6.E−04	1.3.E+02	− 0.2	2.9.E−03	2.4.E+03	SH3BP4	SH3 domain binding protein 4
V6	chr4: 71122281	rs13146558	G/T	0.24	0.22	0.37	0.41	− 0.4	8.4.E−06	6.7.E+00	− 0.44	7.5.E−04	6.0.E+02	− 0.23	3.1.E−03	2.5.E+03	CABS1	Calcium binding protein, spermatid associated 1
**V6**	**chr6: 16481725**	**rs2237192**	**T/G**	**0.18**	**0.21**	**0.07**	**0.02**	**− 0.44**	**7.3.E**−**06**	5.8.E+00	**− 0.58**	**4.0.E**−**05**	3.2.E+01	**− 0.17**	**3.7.E**−**02**	3.0.E+04	**ATXN1**	**Ataxin 1**
V6	chr11: 44017916	rs11037789	T/G	0.28	0.39	0.06	0.15	− 0.37	9.2.E−06	7.3.E+00	− 0.53	3.9.E−05	3.1.E+01	− 0.13	4.0.E−02	3.2.E+04	ACCSL	1-aminocyclopropane-1-carboxylate synthase homolog like
V7	chr1: 242381187	rs76072862	A/G	0.11	0.12	0.1	0.11	− 0.69	3.3.E−06	2.6.E+00	− 0.64	3.4.E−03	2.7.E+03	− 0.81	5.6.E−07	4.5.E−01	PLD5	Phospholipase D family member 5
V7	chr4: 140609325	rs1377382	G/A	0.19	0.21	0.02	0.02	− 0.48	9.5.E−06	7.6.E+00	− 0.46	2.1.E−03	1.7.E+03	− 0.54	5.6.E−05	4.4.E+01	MGST2	Microsomal glutathione S-transferase 2
**V7**	**chr5: 60843706**	**rs6898746**	**A/T**	**0.15**	**0.21**	**0.52**	**0.55**	**− 0.71**	**4.9.E**−**08**	3.9.E−02	**− 0.69**	**1.1.E**−**04**	8.8.E+01	**− 0.73**	**1.1.E**−**05**	8.6.E+00	**ZSWIM6**	**Zinc Finger SWIM-type containing 6**
V7	chr7: 81757727	rs258692	A/G	0.43	0.44	0.25	0.3	− 0.45	5.9.E−06	4.7.E+00	− 0.49	3.4.E−04	2.7.E+02	− 0.28	2.6.E−02	2.0.E+04	CACNA2D1	Calcium voltage-gated channel auxiliary subunit alpha2delta 1
V7	chr9: 106013715	rs7041631	C/A	0.21	0.21	0.59	0.36	− 0.54	1.3.E−06	1.0.E+00	− 0.64	3.3.E−05	2.6.E+01	− 0.37	8.8.E−03	7.0.E+03	LINC01492	Long intergenic non-protein coding RNA 1492
V7	chr11: 122790097	rs58906513	G/C	0.2	0.2	0.06	0.1	− 0.53	3.1.E−06	2.4.E+00	− 0.69	1.1.E−05	9.0.E+00	− 0.28	4.2.E−02	3.4.E+04	C11orf63	Junctional cadherin complex regulator
V7	chr12: 54127513	rs7131877	T/C	0.83	0.75	0.36	0.37	− 0.59	1.4.E−06	1.2.E+00	− 0.62	2.4.E−04	1.9.E+02	− 0.47	3.5.E−03	2.8.E+03	CISTR	Chondrogenesis-associated transcript
V7	chr22: 28083520	rs134119	A/G	0.42	0.39	0.5	0.43	− 0.47	2.0.E−06	1.6.E+00	− 0.66	2.0.E−06	1.6.E+00	− 0.26	3.3.E−02	2.7.E+04	MN1	MN1 proto-oncogene, transcriptional regulator
V8	chr3: 151738736	rs6800439	G/A	0.3	0.3	0.58	0.46	− 0.51	5.0.E−06	4.0.E+00	− 0.51	7.7.E−04	6.2.E+02	− 0.27	3.5.E−02	2.8.E+04	AADACL2	Arylacetamide deacetylase like 2
**V8**	**chr4: 71122281**	**rs13146558**	**G/T**	**0.24**	**0.22**	**0.37**	**0.41**	**− 0.57**	**3.5.E**−**07**	2.8.E−01	**− 0.57**	**2.4.E**−**04**	1.9.E+02	**− 0.41**	**3.1.E**−**04**	2.5.E+02	**CSN3**	**Calcium Binding protein, spermatid associated 1**
V8	chr7: 18953422	rs34574947	T/C	0.19	0.21	0.33	0.23	− 0.54	7.9.E−06	6.3.E+00	− 0.55	9.1.E−04	7.3.E+02	− 0.38	3.5.E−03	2.8.E+03	HDAC9	Histone deacetylase 9
V9	chr1: 242754990	rs10803057	T/C	0.29	0.37	0.31	0.38	− 0.47	9.9.E−06	7.9.E+00	− 0.47	4.5.E−04	3.6.E+02	− 0.46	9.6.E−03	7.7.E+03	PLD5	Phospholipase D family member 5
V9	chr2: 220226855	rs117643098	A/G	0.15	0.08	0	0	− 0.72	5.7.E−06	4.5.E+00	− 0.79	1.4.E−04	1.2.E+02	− 0.6	1.3.E−02	1.1.E+04	RESP18	Regulated endocrine specific protein 18
V9	chr6: 120999930	rs7756766	C/T	0.13	0.11	0.01	0.06	− 0.76	2.0.E−07	1.6.E−01	− 0.69	2.0.E−04	1.6.E+02	− 0.9	1.9.E−04	1.5.E+02	TBC1D32	TBC1 domain family member 32
V9	chr8: 89204779	rs12544601	A/G	0.12	0.06	0	0.12	− 0.69	8.6.E−06	6.8.E+00	− 0.72	4.1.E−04	3.3.E+02	− 0.65	7.3.E−03	5.8.E+03	MMP16	Matrix metallopeptidase 16
**V9**	**chr10: 67974293**	**rs10762029**	**T/C**	**0.35**	**0.41**	**0.47**	**0.5**	**− 0.51**	**1.5.E**−**06**	1.2.E+00	**− 0.43**	**2.4.E**−**03**	1.9.E+03	**− 0.64**	**4.7.E**−**05**	3.8.E+01	**CTNNA3**	**Casein Kappa**
V9	chr20: 11958082	rs6134419	C/T	0.19	0.23	0.06	0.04	− 0.61	4.5.E−06	3.6.E+00	− 0.55	9.8.E−04	7.8.E+02	− 0.73	9.1.E−04	7.3.E+02	BTBD3	BTB domain containing 3
V10	chr3: 30833647	rs6763790	T/C	0.14	0.16	0.03	0.02	− 0.76	1.2.E−06	9.5.E−01	− 0.71	2.2.E−04	1.7.E+02	− 0.81	3.6.E−03	2.8.E+03	GADL1	Glutamate decarboxylase like 1
V10	chr5: 178763160	rs56105546	A/G	0.18	0.22	0.28	0.29	− 0.64	1.2.E−06	9.6.E−01	− 0.56	1.3.E−03	1.0.E+03	− 0.76	1.8.E−04	1.5.E+02	ADAMTS2	ADAM metallopeptidase with thrombospondin type 1 motif 2
V10	chr6: 160982051	rs10945677	T/C	0.43	0.41	0.22	0.15	0.55	3.8.E−07	3.0.E−01	0.57	3.7.E−05	2.9.E+01	0.5	3.2.E−03	2.5.E+03	LPA	Lipoprotein(A)
V10	chr6: 32797684	rs241437	G/A	0.53	0.54	0.39	0.4	0.48	2.1.E−06	1.7.E+00	0.43	1.1.E−03	8.5.E+02	0.57	3.0.E−04	2.4.E+02	TAP2	Transporter 2, ATP binding cassette subfamily B member
V10	chr6: 32685865	rs9275653	A/G	0.75	0.76	0.64	0.6	0.59	4.8.E−06	3.8.E+00	0.46	4.8.E−03	3.9.E+03	0.83	9.8.E−05	7.9.E+01	HLA-DQA2	Major histocompatibility complex, class II, DQ alpha 2
V10	chr9: 29641836	rs541535	A/T	0.22	0.21	0.36	0.34	0.6	9.7.E−06	7.8.E+00	0.71	7.4.E−05	5.9.E+01	0.4	4.9.E−02	3.9.E+04	LINGO2	Leucine rich repeat and Ig domain containing 2
V10	chr10: 37753415	rs2505679	A/G	0.18	0.18	0.22	0.21	− 0.61	4.0.E−06	3.2.E+00	− 0.6	2.9.E−04	2.3.E+02	− 0.59	8.7.E−03	6.9.E+03	MTRNR2L7	MT-RNR2 Like 7
V10	chr12: 5682166	rs10492185	T/C	0.18	0.2	0.28	0.34	− 0.65	4.7.E−06	3.8.E+00	− 0.62	8.6.E−04	6.9.E+02	− 0.69	1.8.E−03	1.4.E+03	ANO2	Anoctamin 2
V11	chr2: 16266203	rs144460588	G/A	0.57	0.53	0.88	0.83	0.57	4.3.E−06	3.4.E+00	0.5	1.9.E−03	1.5.E+03	0.65	1.1.E−03	8.8.E+02	FAM49A	CYFIP related Rac1 interactor A
**V11**	**chr9: 119621796**	**rs6478258**	**A/G**	**0.28**	**0.26**	**0.27**	**0.29**	**− 0.63**	**3.0.E**−**06**	2.4.E+00	**− 0.66**	**8.4.E**−**05**	6.7.E+01	**− 0.58**	**1.1.E**−**02**	8.8.E+03	**ASTN2**	**Astrotactin 2**
V11	chr10: 92301707	rs11816911	A/G	0.12	0.16	0.12	0.07	− 0.95	7.1.E−08	5.7.E−02	− 1.1	1.4.E−06	1.1.E+00	− 0.7	1.1.E−02	9.1.E+03	LOC101926942	Uncharacterized LOC101926942
V11	chr11: 110227924	rs11213366	T/C	0.23	0.25	0.25	0.37	− 0.66	3.7.E−06	3.0.E+00	− 0.64	2.7.E−04	2.2.E+02	− 0.69	5.1.E−03	4.1.E+03	LOC105369486	Uncharacterized LOC105369486
V11	chr13: 82179123	rs7333519	T/A	0.34	0.32	0.42	0.6	0.53	8.3.E−06	6.6.E+00	0.56	2.6.E−04	2.1.E+02	0.45	2.0.E−02	1.6.E+04	SLITRK1	SLIT and NTRK like family member 1
**V11**	**chr18: 9029690**	**rs149280597**	**A/G**	**0.32**	**0.27**	**0.05**	**0.04**	**− 0.57**	**9.2.E**−**06**	7.3.E+00	**− 0.59**	**3.1.E**−**04**	2.5.E+02	**− 0.5**	**1.6.E**−**02**	1.3.E+04	**MTCL1**	**Microtubule Crosslinking factor 1**

Significant values are in bold.

*Physical positions are based on NCBI build 37 of the human genome.

The SNPs of the existing Facial Measurement GWAS results were checked once more in the results of this study, and the results were included in a separate Supplementary Table S5. We could test 20 SNPs that was previously reported in other studies, and described the association results for the early facial growth patterns that the major phenotypes of this study. Most of the tested SNPs were found to have no significance or very weak significance. The reason for this difference is that this study is not an analysis to discover genetic markers related to differences in facial shape between individuals, but an analysis of childhood facial growth patterns.

A limitation of this study was that there were not enough participants and some factors that could affect facial growth were not measured. The facial measurements were appeared 2D images rather than 3D images. To understand for facial growth patterns, we tried to measure for facial traits, such as take a photograph of early age participants in the studio and recruited past pictures. Through like this study, further study will be more understand for facial morphology and facial growth.

## Discussion

Human development is characterized by distinct developmental processes, especially during adolescence, and the speed and direction of craniofacial development differ for each person. Therefore, under the assumption that the speed and direction of development would differ across individuals, we calculated the changes in facial measurements according to age over time for each individual and for each indicator and divided these change patterns into five major categories. An index was calculated based on the positioning of landmarks in the facial profile picture, according to a widely used method for current facial analyses. GWAS analysis of facial development patterns was performed by recoding each of the five growth patterns as individual values.

Despite the significant difference from the existing approach such as GWAS analysis of face measurements or facial deformities, we were able to obtain GWAS results that were repeatedly associated with facial features. A total of 97 significant indicators were identified, including indicators related to craniofacial development in 19 areas. Because the probabilistic effects and differences of facial phenotypes must be confirmed by replication analysis of identified loci, we divided the collection into two groups, and the genetic influences in each group were analyzed for stochastic effects through replication analysis and it were confirmed through the replication indicators.

In the nose, the width of the nostrils most commonly increased with age, and the length of the nose showed the largest tendency toward increase across all of the vertical axis. Around the lips, many individuals showed patterns of increase or maintenance, whereas the patterns associated with the mouth were distributed differently in each population, indicating the degree of difference in the growth patterns among individuals.

The length of H1 and H2 have the most decreasing patterns with aging. H5 and H6 have the most increasing patterns with aging. The most noticeable changes were observed for the cutis and subcutaneous bone.

For both POP1 and POP2, as shown in Table 1, H3 most commonly have the most increasing patterns, whereas H4 most commonly have the most decreasing patterns. Because as they grow, the elasticity of adjacent tissues under the eyes decreases due to growth, resulting in the sagging tail appearance of the eyes. In addition, H8, H9, and H10 appeared to be very significant indicators, which appeared to influence each other. H8 is a diagonal length on the left side of the nose, which tends to increase with age. H9 is the width of the nose, which tends to increase with age because the lower lateral cartilage and the skin surrounding the ends of the septum weaken, losing elasticity.

Among the vertical axis lengths, many indicators showed similar patterns of increase as the horizontal area, and the vertical axis indicators of the face appear to affect each other during growth. The vertical lengths of the eyes, V1 and V2, showed the greatest tendency toward an increasing pattern. The length of the nose, measured by V5–V8, commonly increases until the age of 20 years. The characteristics of the nose are well known and include major changes, such as long, drooping tips^19^. The bone base that supports the nose in youth, a pair of nasal bones, and the ascending process of the maxilla are responsible for many of the soft tissue changes that are observed in the nose during aging^20^.

The pattern frequencies measured for EAFG showed that although we used independent populations, our results were replicated in each population. As shown in Table 1, approximately 70% of the facial development patterns were replicated in each group. The index with the highest frequency was replicated in each group, indicating a common pattern across populations. Although a few indices showed a different pattern, these unique indices clustered into large categories (increase or decrease).

However, no analysis model exists for facial growth, and the classification of facial growth as a visual expression clustering model is limiting. In this study, we analyzed by applying the –assoc option provided by PLINK software, and the results of this analysis are based on statistical models called likelihood ratio test and Wald test. The reason for applying this analysis is that the phenotype we are targeting is not a general quantitative phenotype, but multinomial variables called facial growth pattern. The currently available method for genome-wide analysis of these variables and multiple SNPs was the statistical model provided by PLINK software. Therefore, the significance between the SNP and the phenotype discovered in this study can be understood as an analysis result of whether the SNP has the explanatory power to explain the phenotype. Some genetic studies based on multinomial variables, and among them, we can check an example of applying the same likelihood ratio test as ours^21,22^.

In addition, the number of samples cannot be considered representative of all Koreans. However, this study represents the first attempt to classify the pattern of facial growth, and when data from two independent groups collected at the same time are analyzed and compared, the common result (the frequency of the pattern is more than 70% coincident) overcomes these limitations.

Most facial changes occur before age 18, but growth and facial remodeling have been shown to continue throughout life. The facial skeleton is generally believed to expand continuously throughout life^23^, which is reflected in the gradual increase in certain facial anthropometric measurements with age, such as anterior nasal cavity and facial width. Certain measurements increase significantly with aging, but some measurements are reduced. The chin length becomes shorter as the mandible of the face grows backward due to aging, resulting in a shorter overall face length.

Some extrinsic variables like gender^24^, body mass^25^ are known to effect facial morphology. The main influence of gender on facial phenotypes was reported as nasal area and upper facial area, and body mass index (BMI) was reported as a face width characteristic^24^. Obesity-related sites such as cheeks and neck were excluded from the measurement. So, it is thought that the effect of the degree of obesity in this study is relatively small.

**Figure 3:**
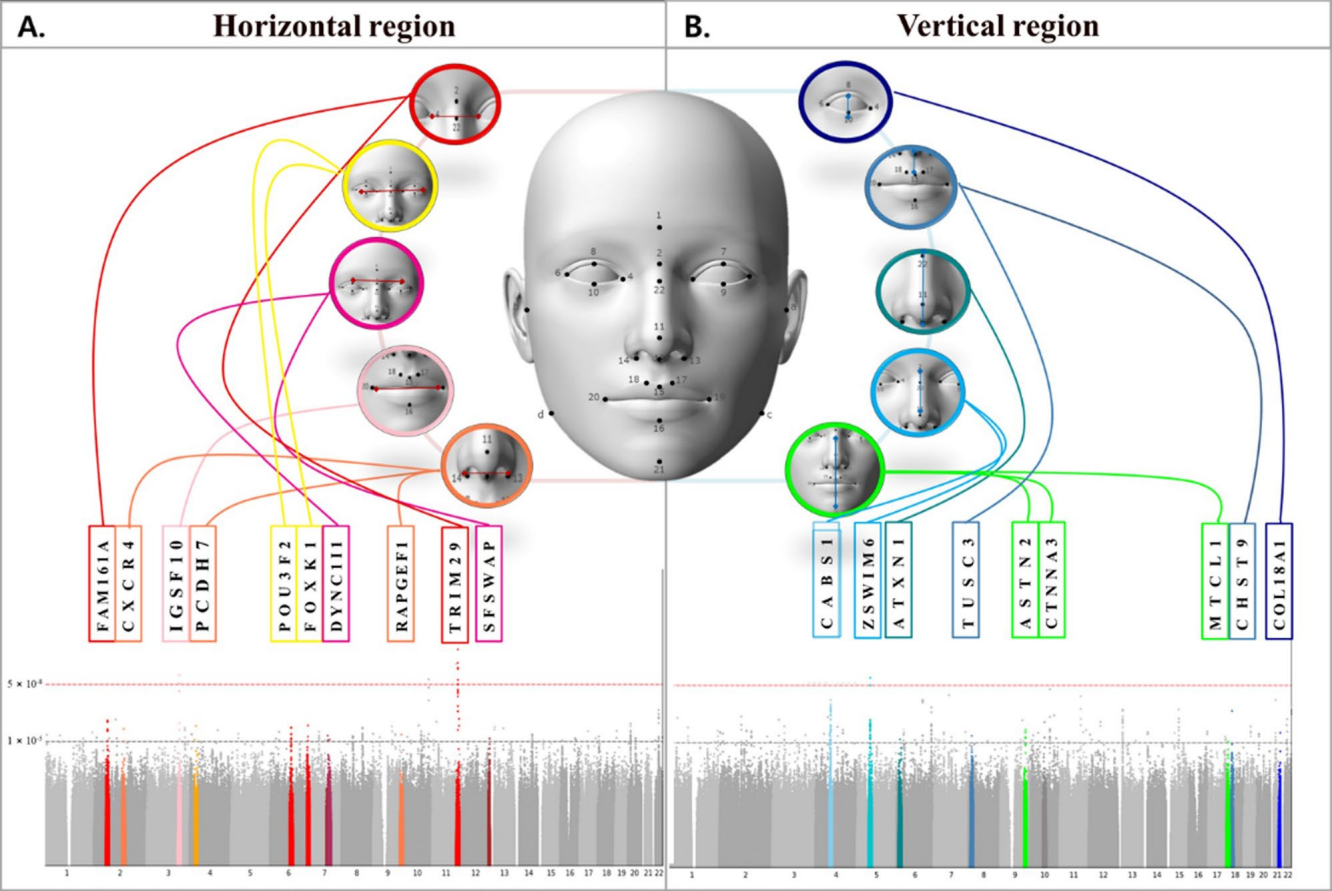
Drawings indicate the facial phenotypes and genes associated with the 5 EAFG patterns identified using genome-wide significant associations.

GWAS results provide a hypothesis-free approach to identifying important genetic variations that underlie craniofacial shape differences within populations^26^. A total of 97 significant or suggestive SNPs in 19 gene regions and loci that have previously been associated with facial morphology were identified in this study. For 19 loci showing significant and suggestive phenotypic associations, substantial literature was identified associating these loci with facial development, as shown in Fig. 3. In the current work, we found 10 suggestive SNPs in the horizontal region: *FOXK1*^27^, *IGSF10*^28^, *FAM161A*^29^, *POU3F2*^30^, *DYNC111*^31^, *SFSWAP*^32^, *TRIM29*^33^, *RAPGEF1*^34^, *PCDH7*^35^, and *CXCR4*^36^. We also found 9 suggestive SNPs in the vertical region: *ZSWIM6*^37^, *CSN3*^38^, *ATXN1*^39^, *COL18A1*^40^, *CHST9*^41^, *CTNNA3*^42^, *ASTN2*^43^, *TUSC3*^44^, and *MTCL1*^45^. The gene annotations from the UCSC database (https://genome.ucsc.edu) was used to predict the functional effects of the variants. The genes reported to affect embryonic development from UCSC database included *CXCR4*^46^ in the horizontal region and *CSN3*^38^ and *TUSC3*^44^ in the vertical region. The genes reported to affect cranial growth and brain development included *ZSWIM6*, *ATXN1*^38^, *ASTN2*^43^, and *MTCL1*^45^ in the vertical region. In addition, genes related to molecular mechanisms in the regulation of skeletal muscle and cartilage included *FOXK1*^27^, *RAPGEF1*^34^, and *IGSF10*^28^ in the horizontal region, and *COL18A1*^40^ and *CHST9*^41^ in the vertical region. The genes associated with retinal circuit components and the growth of sensory organs were *FAM161A*^29^, *POU3F2*^30^, and *SFSWAP*^32^ in the horizontal region. Finally, the genes related to frontonasal and dysmorphic facial features were *PCDH7*^35^, *TRIM29*^33^, and *DYNC111*^31^ in the horizontal region and *CTNNA3*^42^ in the vertical region.

The authors have generated an interesting report using 2 dimensional images to perform a facial shape GWAS study focused not on fixed time points, but on ontogenetic growth trajectories. To my knowledge, this is a novel analysis. It is also performed in an interesting way, using a mixture of photo types. The argument for this paper is framed around using this for the purpose of being able to improve the accuracy of age progression for missing children, but there is very little discussion of that and this is a very intriguing basic science question.

The facial growth patterns that occur in childhood remain poorly understood and estimating facial growth simply through photographic analysis or the use of existing facial indicators can be difficult. In addition, an individual’s unique facial morphologies can be difficult to quantify using simple photographic indicator analysis. The characteristics of facial growth should consider differences in each individual’s innate genetic makeup. This study is meaningful because we have classified and characterized facial growth patterns that occur in childhood and will contribute to research on face growth, face recognition, and potentially contribute to finding missing children in the future. This study can serve as a basis for understanding facial morphology and can be expanded to various research fields exploring facial growth, including forensic sciences for both adults and children.

## Materials and methods

### Study participants

The facial images were obtained from the Human ICT, a company specializing in collecting face data. Related individuals among the participants were not included in the analysis. The facial measurement data were obtained from the National Project of the Missing Child conducted by the Korea Institute of Science and Technology (KIST). Two independent populations (POP1 and POP2) between the ages of 18 and 20 were recruited in different periods: 172 individuals in POP1 were recruited from January 2019 to July 2020, and 100 individuals in POP2 were recruited from July 2020 to September 2020. Relatives were not included, only individuals were included. Two types of facial photos were collected: a current picture and older pictures of the same individual. Each individual’s current photo was taken in a studio using a Canon EOS 1300D camera with 2592 × 1728 resolution at 400 lx illumination. The participants were asked to close their mouths, hold their faces in a neutral expression, and prevent hair from covering their foreheads. The older photos comprised various ages that can constitute individual chronologies, and a minimum of 4 to a maximum of 21 photos were collected from each participant. Among older facial photos, high-quality photos, such as passport photos and school graduation photos, were prioritized. We also asked the participants to supply photos in which the face was in a neutral expression, not family photos, and torn photos or those that were creased over the face were excluded. Age information was collected for each of the past photos. All facial photographs were digitized with a scanner. We collected a total of 172 current photos and 884 past photos for POP1 and 100 current photos and 600 past photos for POP2.

### Ethics statement

The research was conducted in accordance with the principles described in the Declaration of Helsinki^46^. The Institutional Review Board of Theragen Bio Institute approved this study (internal review board No.: 700062-20181130-GP-006-01), and all participants provided written informed consent.

### Craniofacial measurements

To extract features for craniofacial measurements in current and past facial photographs, we first detected the facial region in each photograph using Dlib^47^. Dlib^47^ detects the face and automatically identifies facial landmarks after the facial region is detected. Among the numerous facial feature points detected, we selected 19 feature points and utilized two facial landmark detectors to automatically extract those feature points, as shown in Fig. 1. One of the detectors was used to extract the facial landmarks using an hourglass network-based feature adaptation network (FAN)^48^ approach, whereas the other detector was an in-house program that combined Dlib^47^ and Stasm^49^. The FAN method stacked three hourglass networks, including residual architecture, which is parallel, hierarchical, and multi-scale blocks^50^, to enhance the performance of the feature localization. Stasm^50^ is an active shape model (ASM)^51^ method with feature descriptors that we fused to the Dlib^47^ program. All detectors were programmed in C++ and python in a Qt environment. Facial detection and feature point extraction were performed as automatic processes but could also be manually modified to obtain more accurate feature locations by a well-trained operator (accuracy: 98.8% on average).

Euclidean distances between two selected points based on the 19 selected features were calculated, as shown in Supplementary Table 2. A total of 21 facial metric values were calculated. Before this process, the distances between the centers of both eyes were normalized to 1 for all images to avoid issues associated with scale differences between components and differences in the Z-axis between the subjects and the camera. Distance calculation programs were implemented in Visual Studio C++.

### Face measurement quality controls and sample filtering

We clustered measurements into groups of horizontal and vertical measurements and selected 10 phenotypes to represent the horizontal index and 11 phenotypes to represent the vertical index. The 21 facial phenotypes were measured in each current and past profile photograph, using the 19 facial landmarks in Supplementary Table 2. For all facial phenotypes, we performed data quality controls on volunteers, regardless of the trait being studied. We drew boxplot diagrams for each of the 21 measurements to exclude outlier data from the top 2% and the bottom 2% from all past and current measurements using the R programming language used in R packages^52^. Obesity-related sites such as cheeks and neck were excluded from the measurement. An ordinal multinomial model was applied to show that the results were not due to bias. In this study, we analyzed by applying the –assoc option provided by PLINK software, and the results of this analysis are based on statistical models called likelihood ratio test and Wald test^21,22^.

### Early age facial growth pattern (EAFG) analysis

Facial growth shows large variations among individuals; therefore, we graphed the time series of individual facial measurements based on age relative to the current age (Supplementary Fig. S1) using a non-linear model in QQ plot in R package^53^. We defined a total of 5 EAFG growth patterns that were clustered as follows (Fig. 2): Pattern 1 (DD), continued decrease; Pattern 2 (DI), decrease to increase; Pattern 3 (CC), constant; Pattern 4 (ID), increase to decrease, and Pattern 5 (II), continue increased. Among 21 phenotypes, we coded the 5 EAFG patterns and summarized each individual with similar aging trends in Table 1.

### Genotype data

Oral swab samples (KIST) were obtained, and DNA was extracted using ExgeneTM Tissue SV (GeneAll, Seoul, Korea). All DNA samples were amplified and randomly portioned into 25–125-bp fragments, which were purified, resuspended, and hybridized to an Axiom *a*rray (TPMRA chip, Thermo fisher, Seoul, Korea), a customized array based on the Asian Precision Medicine Research Array (Thermo fisher Scientific, Waltham, Massachusetts, USA), Following hybridization, the bound targets were washed under stringent conditions to remove non-specific background to minimize noise resulting from random ligation events. The SNP set was filtered based on genotype call rates (≥ 0.98) and MAF (≥ 0.10). Hardy–Weinberg equilibrium (HWE) was calculated for individual SNPs using an exact test. All SNPs reported in this manuscript demonstrated HWE p-values > 0.0001. After filtering, 560,795 polymorphic SNPs were analyzed on chromosomes 1–22.

### Imputation of SNPs

We conducted an imputation analysis to increase the genome coverage. Imputation of genotypes was performed using minimac4^54^ at the Michigan Imputation Server (MIS) using the 1000G Phase 3 v4 reference panel^20^. We uploaded phased GWAS genotypes and received imputed genomes in return. After imputation, 7,375,270 polymorphic SNPs were analyzed on chromosomes 1–22. INFO score is over than 0.8.

### Genome-wide association studies of EAFG patterns

To identify not only individual indicators but also indicators that commonly affect facial growth, we performed an analysis that combined the phenotypes of POP1 and POP2. We also conducted a genome-wide association scan of the coded 1 to 5 EAFG growth patterns using asymptotic analyses (likelihood ratio test and Wald test) using the combined population of POP1 and POP2. Population-specific and combined population analyses were performed using PLINK version 1.9 (https://www.cog-genomics.org/plink/)^55^, SPSS (IBM SPSS Statistics Inc., New York, U.S.)^56^, and R Statistical Software^52^. We calculated the beta coefficient and the standard error (SE) values for the association study. To compare the GWAS results for each population, we conducted a replication study using 172 samples from POP1 and 100 samples from POP2. We selected the genetic markers associated with the 5 EAFG patterns in each GWAS, determined by association p-values < 1 × 10^−5^ in the combined dataset and p-values < 0.05 for the individual population datasets and the replication study. In this study, GWAS analysis was performed based on about 800,000 SNPs. The Bonferroni correction p-value threshold is applied. The results are shown in Tables 2 and 3 and Manhattan plots are depicted in Supplementary Fig. S3. The QQ plot, generated using R Statistical Software^52^, of the observed p-values showed minimal inflation of the GWAS results from the combined population sample (Supplementary Fig. S2).

### Annotation of SNP-associated genes

To identify and annotate genes that are functionally related to suggestive and significant SNPs identified in the GWAS, SNP locus data were obtained from the UCSC Genome Browser (Genome Bioinformatics Group, University of Santa Cruz, Santa Cruz, CA, USA). The gene annotations from the UCSC database and Genotype-Tissue Expression (GTEx) database (GTEx Analysis Release v.8, http://www.gtexportal.org/) were used to predict the functional effects of the variants. We constructed regional plots of association for regions of interest using the program LocusZoom (Supplementary Fig. S4)^57^.

## Supplementary Information

Supplementary Information.

## Data Availability

Raw genotype or phenotypic data cannot be used due to limitations imposed by ethics. The Summary statistics obtained here are based on the GWAS analysis and can be accessed with the supplementary materials.
