# Supplementary material for: Classification of early age facial growth pattern and identification of the genetic basis in two Korean populations
Source: Sci Rep. 2022 Aug 15;12:13828. doi: 10.1038/s41598-022-18127-6 (PMC9378761; doi:10.1038/s41598-022-18127-6)
Supplement: Supplementary file 1 — Supplementary Information. [file 41598_2022_18127_MOESM1_ESM.zip › Supplementary Figure 1.docx]

**Figure S1. A graph of the time series for individual facial measurements and each individual facial growth pattern.**

1. **H1 (Intercanthal width)**


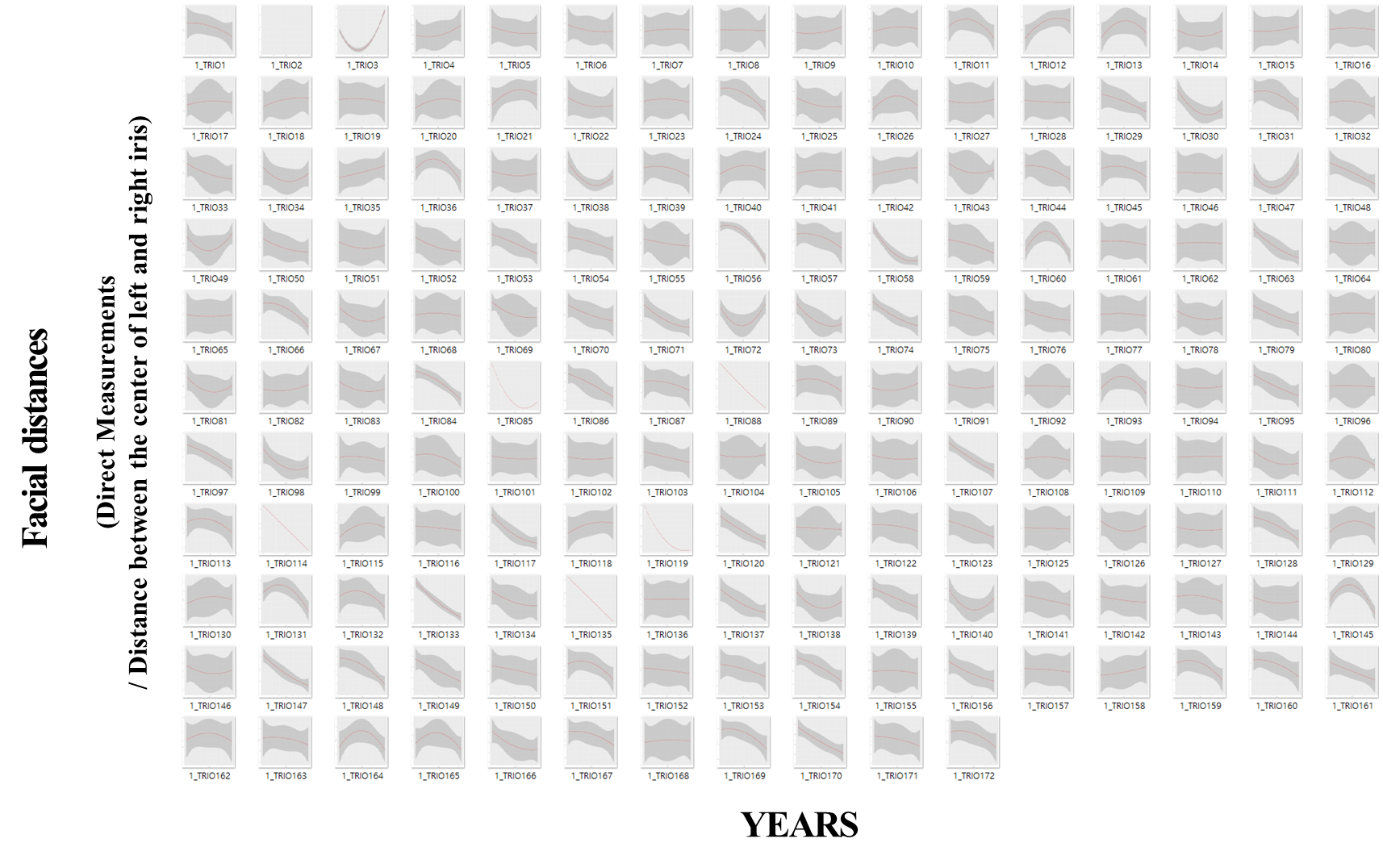


**
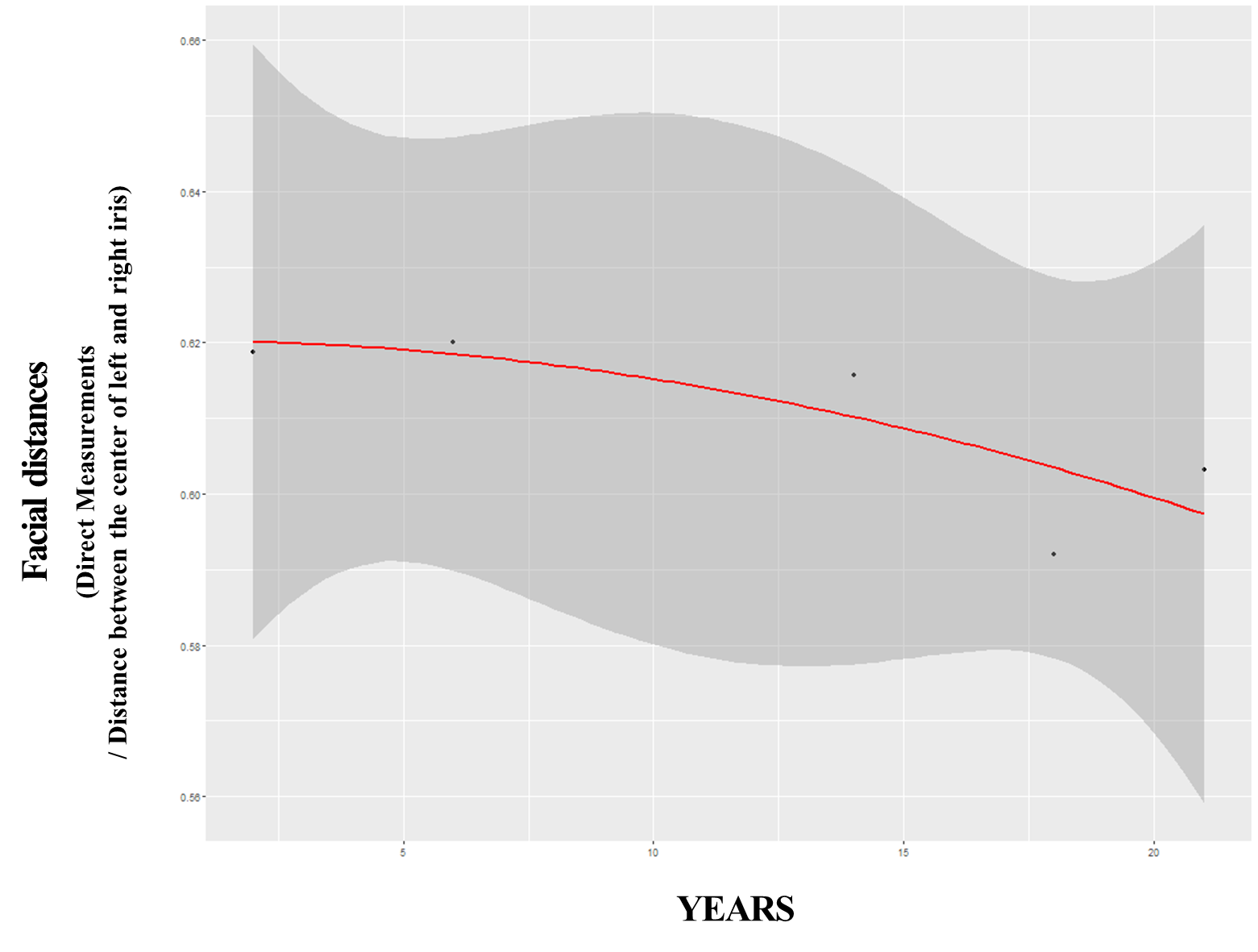
**

**(B) V7 (Nasal bridge height)**

**
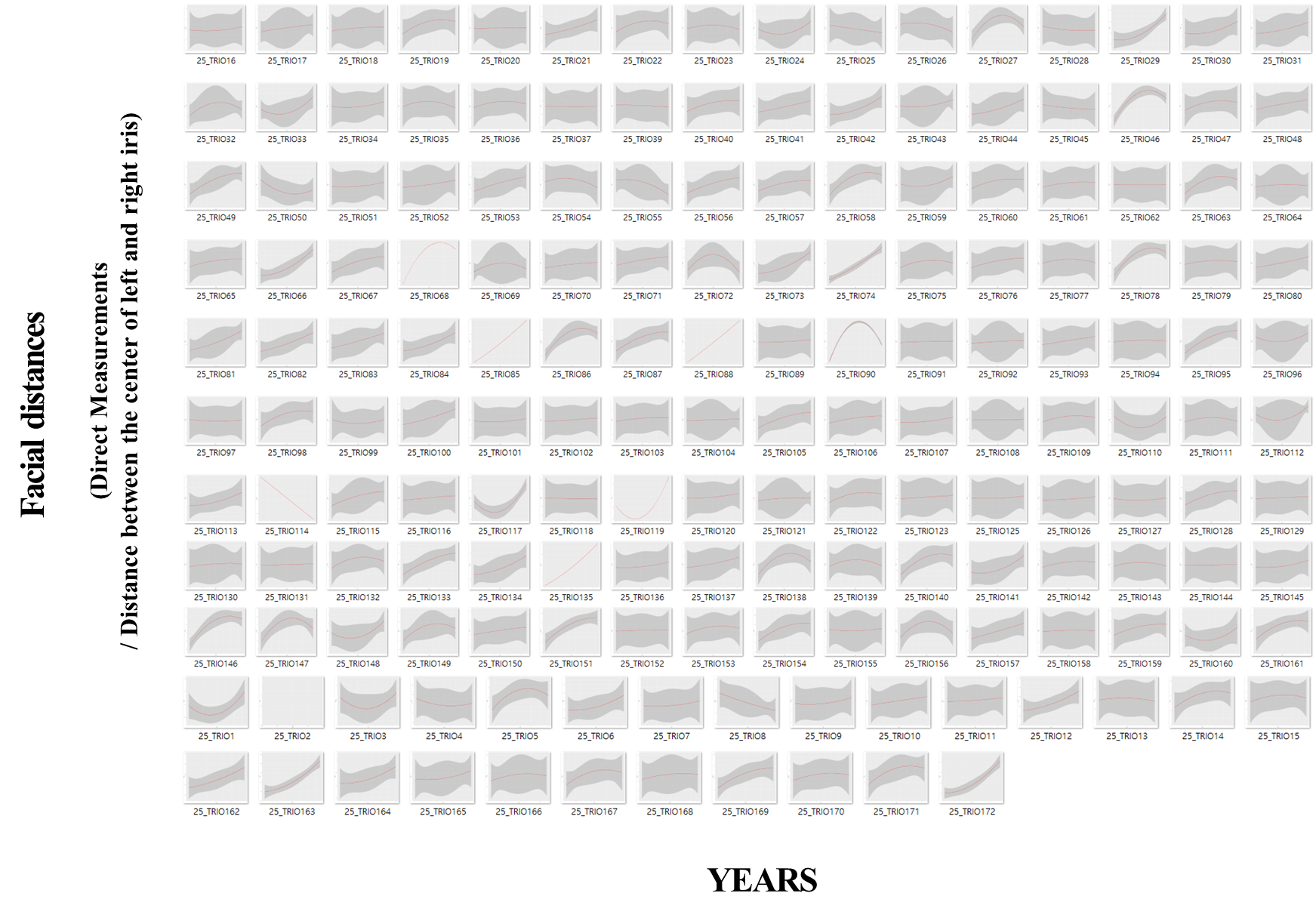
**

**
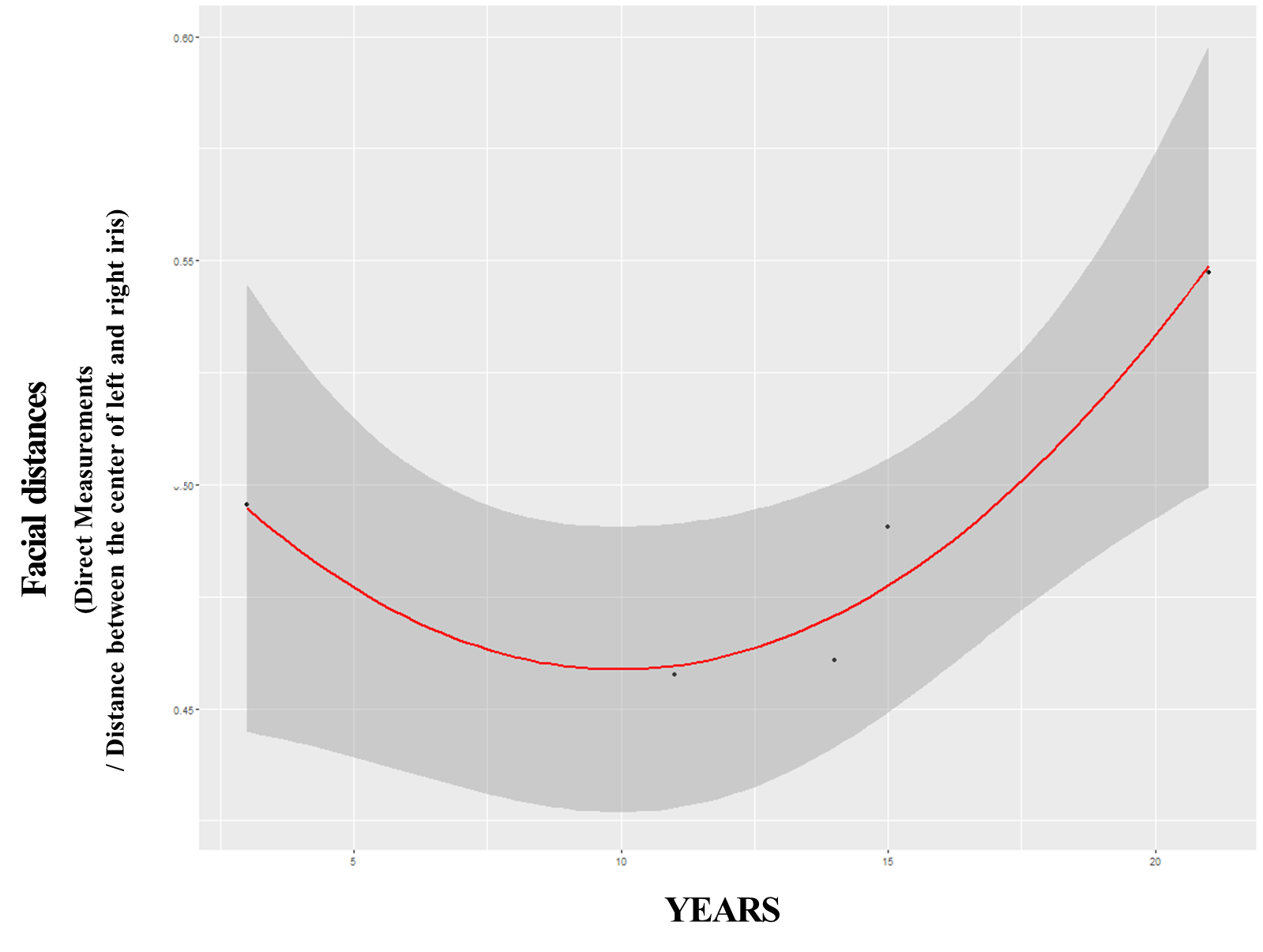
**

**(C) H7 (Mouth width)**


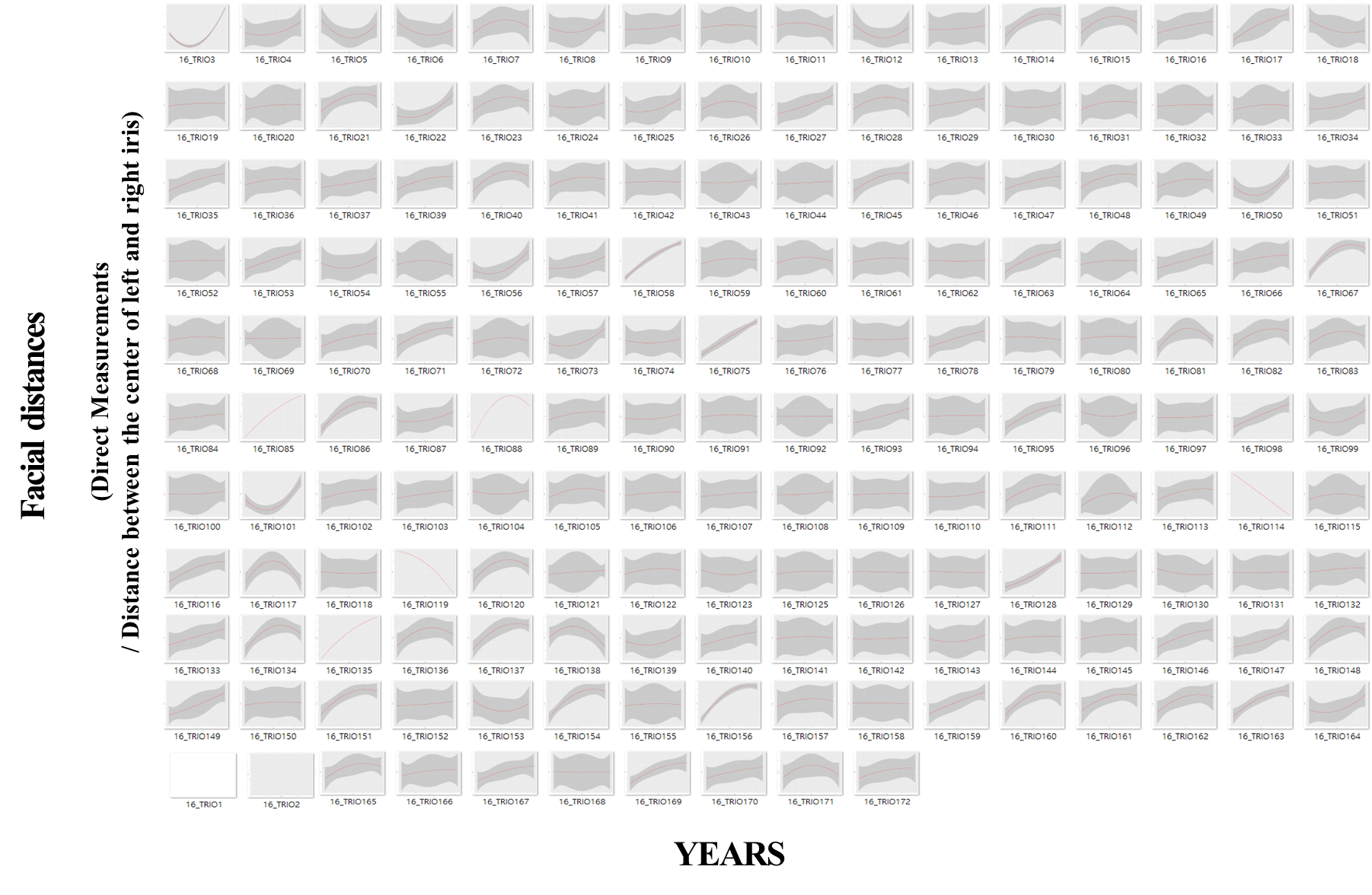


**
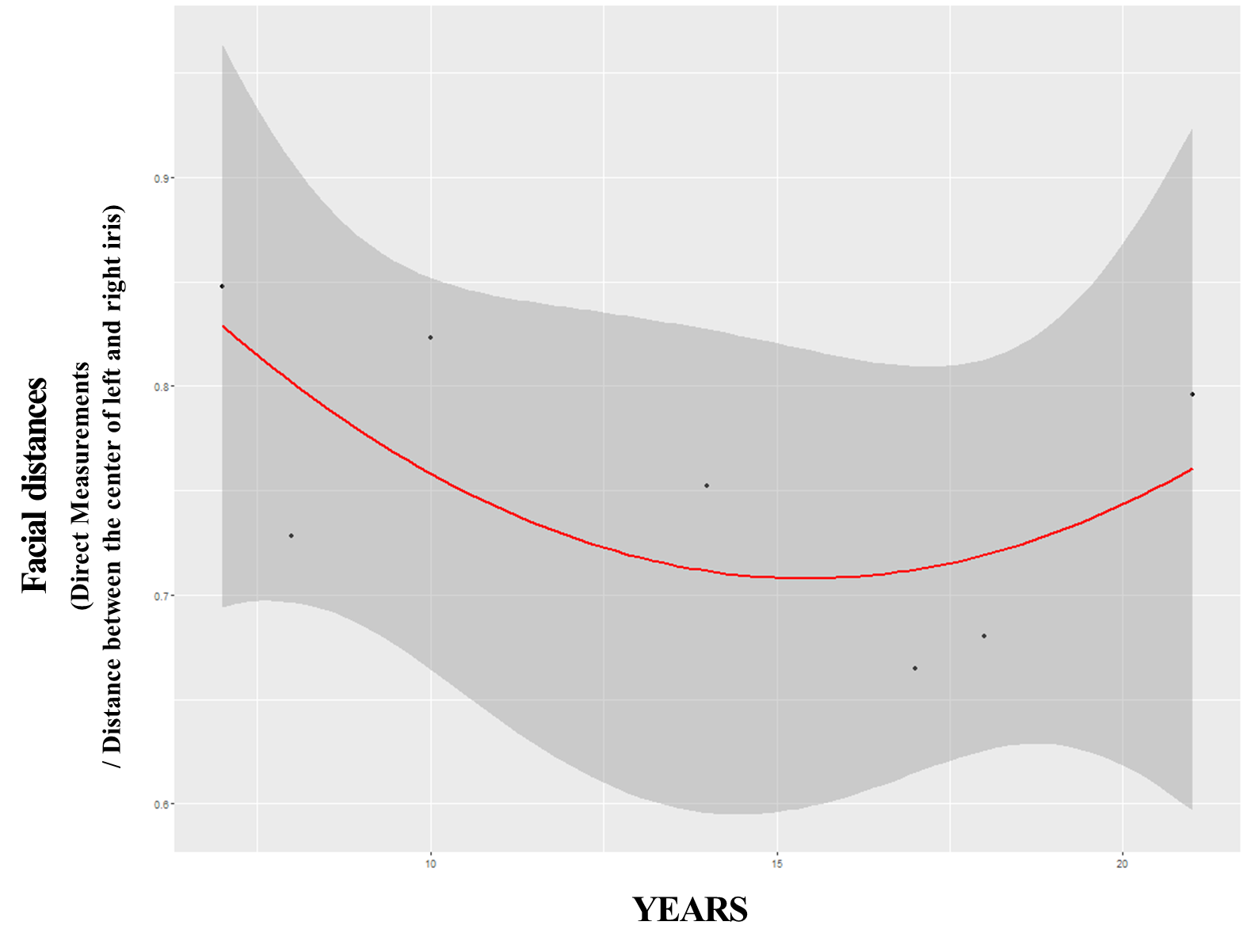
**
