# Supplementary material for: Classification of early age facial growth pattern and identification of the genetic basis in two Korean populations
Source: Sci Rep. 2022 Aug 15;12:13828. doi: 10.1038/s41598-022-18127-6 (PMC9378761; doi:10.1038/s41598-022-18127-6)
Supplement: Supplementary file 1 — Supplementary Information. [file 41598_2022_18127_MOESM1_ESM.zip › Supplementary Figure 3.docx]

**Figure S3. Manhattan plot of the genome-wide association study p-values for the combined analysis of 21 phenotypes. Associations were assessed using the Wald test**

**
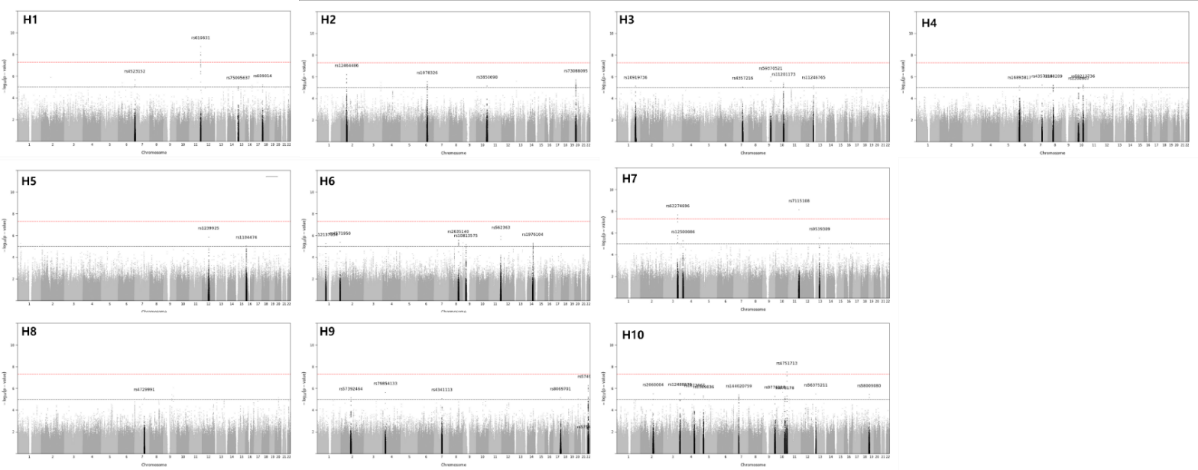

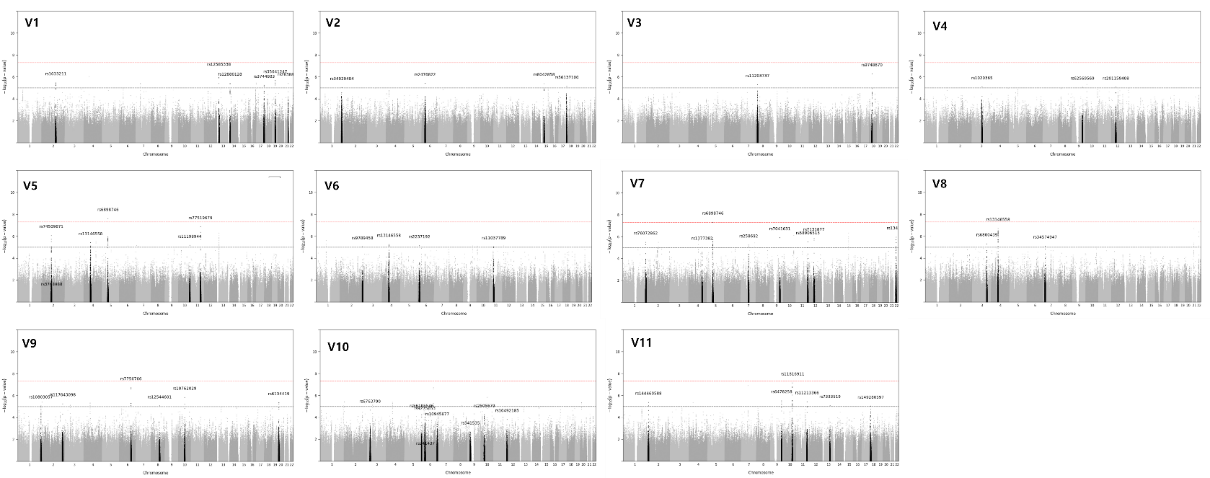
**
