# Supplementary material for: Classification of early age facial growth pattern and identification of the genetic basis in two Korean populations
Source: Sci Rep. 2022 Aug 15;12:13828. doi: 10.1038/s41598-022-18127-6 (PMC9378761; doi:10.1038/s41598-022-18127-6)
Supplement: Supplementary file 1 — Supplementary Information. [file 41598_2022_18127_MOESM1_ESM.zip › Supplementary Figure 4.pptx]

## Slide 1
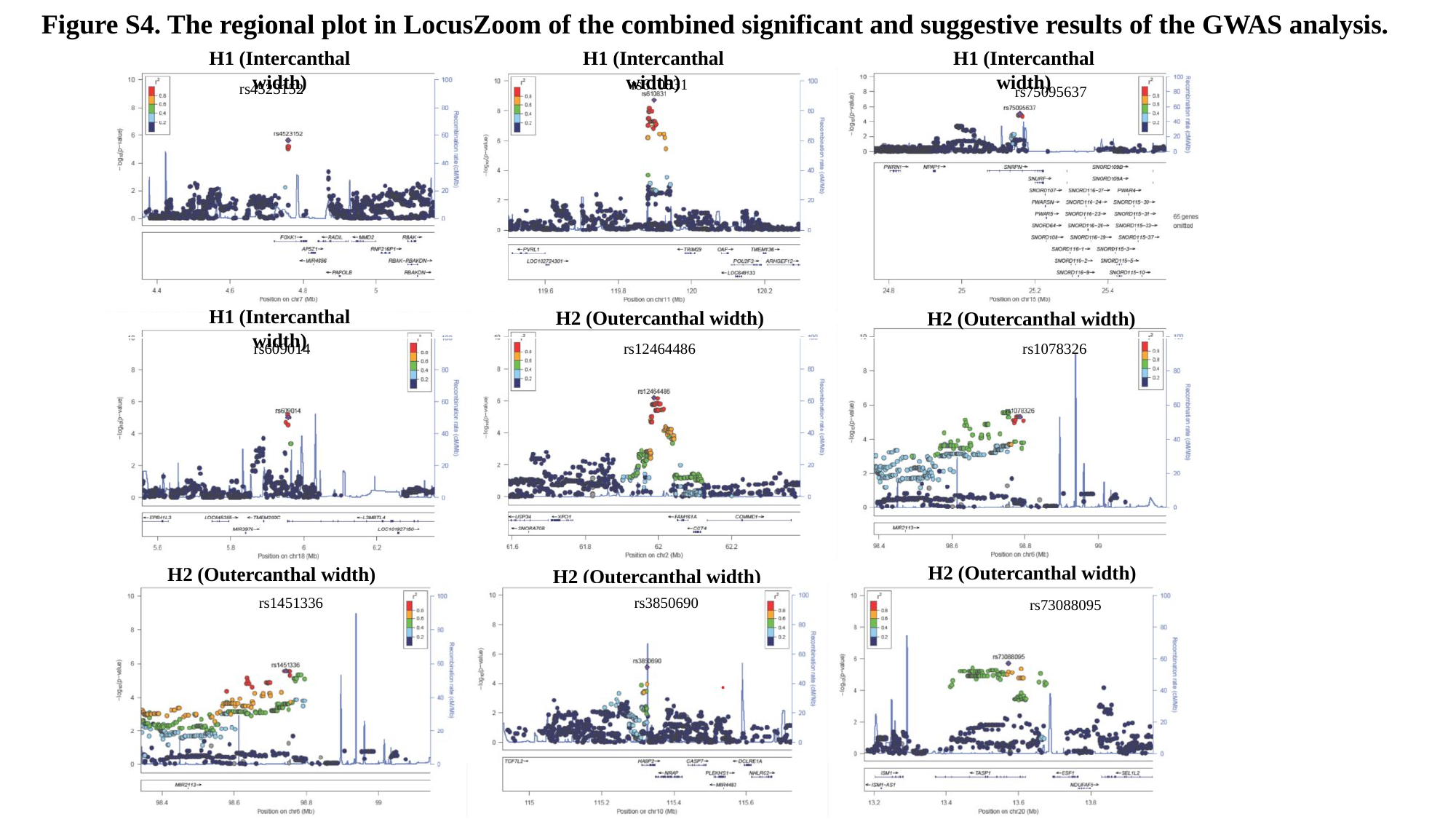

Figure S4. The regional plot in LocusZoom of the combined significant and suggestive results of the GWAS analysis.
H1 (Intercanthal width)
H1 (Intercanthal width)
H1 (Intercanthal width)
rs610831
rs4523152
rs75095637
H1 (Intercanthal width)
H2 (Outercanthal width)
H2 (Outercanthal width)
rs1078326
rs609014
rs12464486
H2 (Outercanthal width)
H2 (Outercanthal width)
H2 (Outercanthal width)
rs1451336
rs3850690
rs73088095

## Slide 2
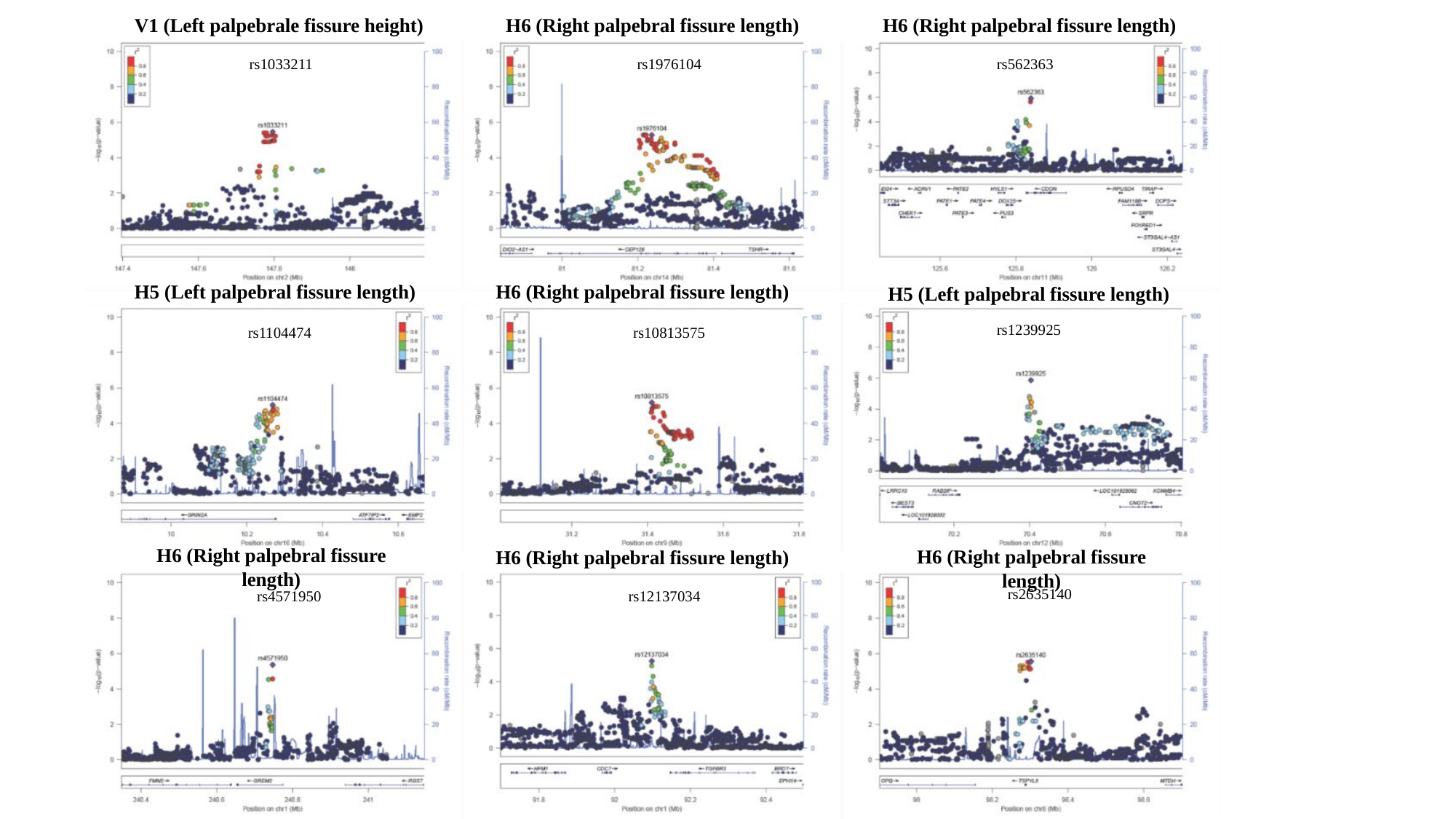

H6 (Right palpebral fissure length)
V1 (Left palpebrale fissure height)
H6 (Right palpebral fissure length)
rs1033211
rs562363
rs1976104
H5 (Left palpebral fissure length)
H6 (Right palpebral fissure length)
H5 (Left palpebral fissure length)
rs1239925
rs1104474
rs10813575
H6 (Right palpebral fissure length)
H6 (Right palpebral fissure length)
H6 (Right palpebral fissure length)
rs2635140
rs4571950
rs12137034

## Slide 3
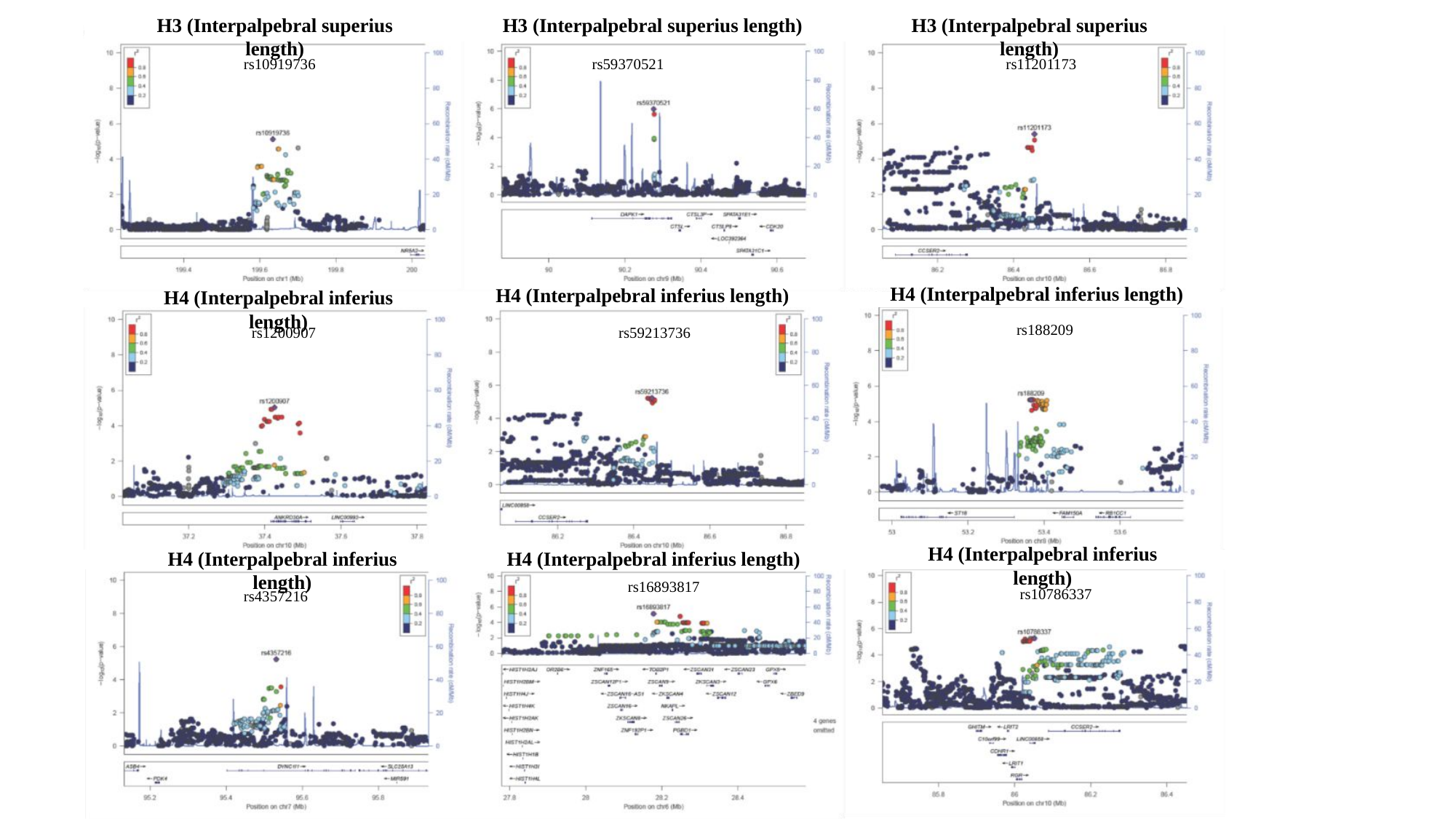

H3 (Interpalpebral superius length)
H3 (Interpalpebral superius length)
H3 (Interpalpebral superius length)
rs10919736
rs11201173
rs59370521
H4 (Interpalpebral inferius length)
H4 (Interpalpebral inferius length)
H4 (Interpalpebral inferius length)
rs188209
rs1200907
rs59213736
H4 (Interpalpebral inferius length)
H4 (Interpalpebral inferius length)
H4 (Interpalpebral inferius length)
rs16893817
rs10786337
rs4357216

## Slide 4
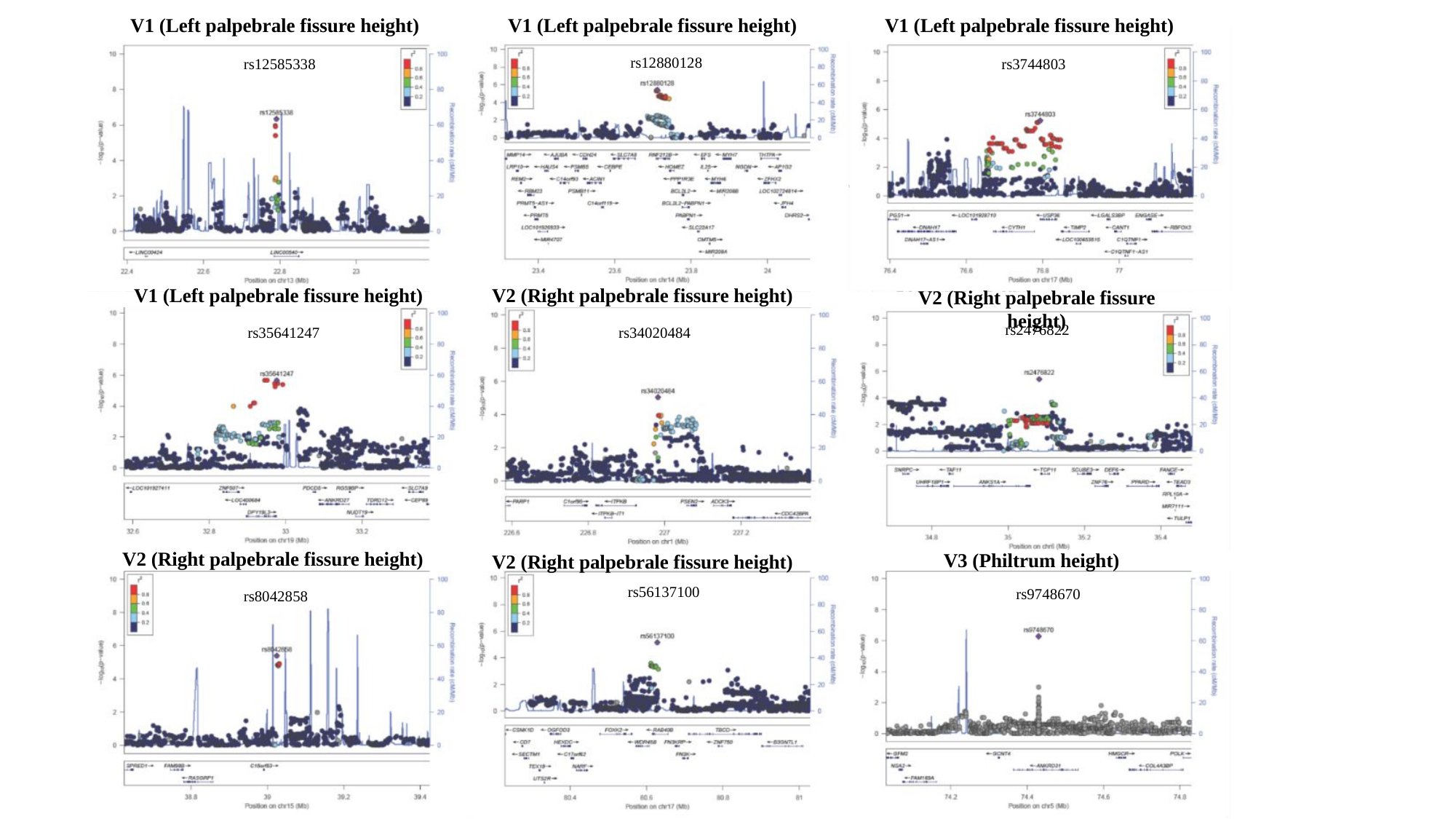

V1 (Left palpebrale fissure height)
V1 (Left palpebrale fissure height)
V1 (Left palpebrale fissure height)
rs12880128
rs12585338
rs3744803
V1 (Left palpebrale fissure height)
V2 (Right palpebrale fissure height)
V2 (Right palpebrale fissure height)
rs2476822
rs35641247
rs34020484
V2 (Right palpebrale fissure height)
V3 (Philtrum height)
V2 (Right palpebrale fissure height)
rs56137100
rs9748670
rs8042858

## Slide 5
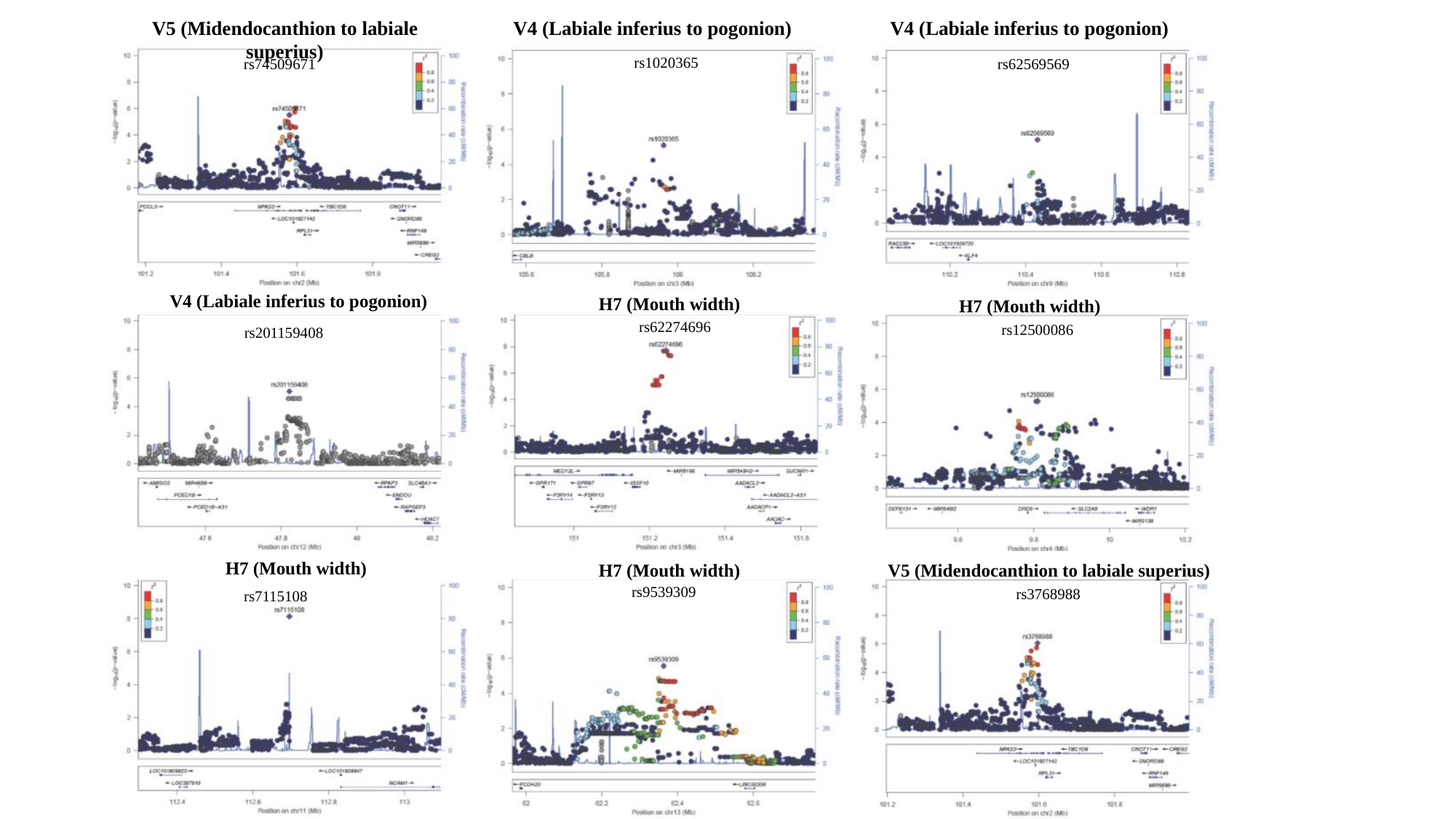

V4 (Labiale inferius to pogonion)
V5 (Midendocanthion to labiale superius)
V4 (Labiale inferius to pogonion)
rs1020365
rs74509671
rs62569569
V4 (Labiale inferius to pogonion)
H7 (Mouth width)
H7 (Mouth width)
rs62274696
rs12500086
rs201159408
H7 (Mouth width)
V5 (Midendocanthion to labiale superius)
H7 (Mouth width)
rs9539309
rs3768988
rs7115108

## Slide 6
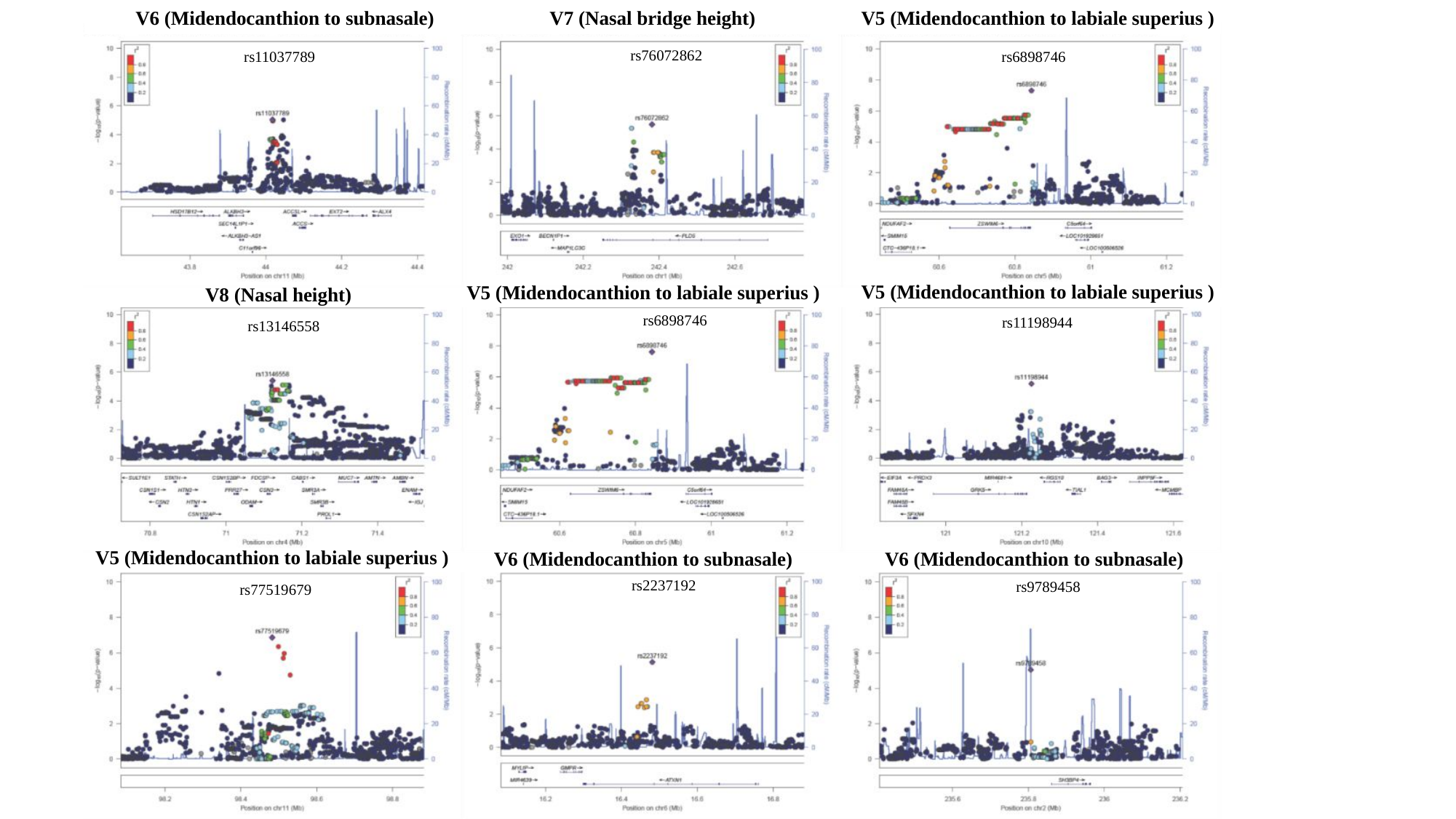

V7 (Nasal bridge height)
V6 (Midendocanthion to subnasale)
V5 (Midendocanthion to labiale superius )
rs76072862
rs11037789
rs6898746
V5 (Midendocanthion to labiale superius )
V5 (Midendocanthion to labiale superius )
V8 (Nasal height)
rs6898746
rs11198944
rs13146558
V5 (Midendocanthion to labiale superius )
V6 (Midendocanthion to subnasale)
V6 (Midendocanthion to subnasale)
rs2237192
rs9789458
rs77519679

## Slide 7
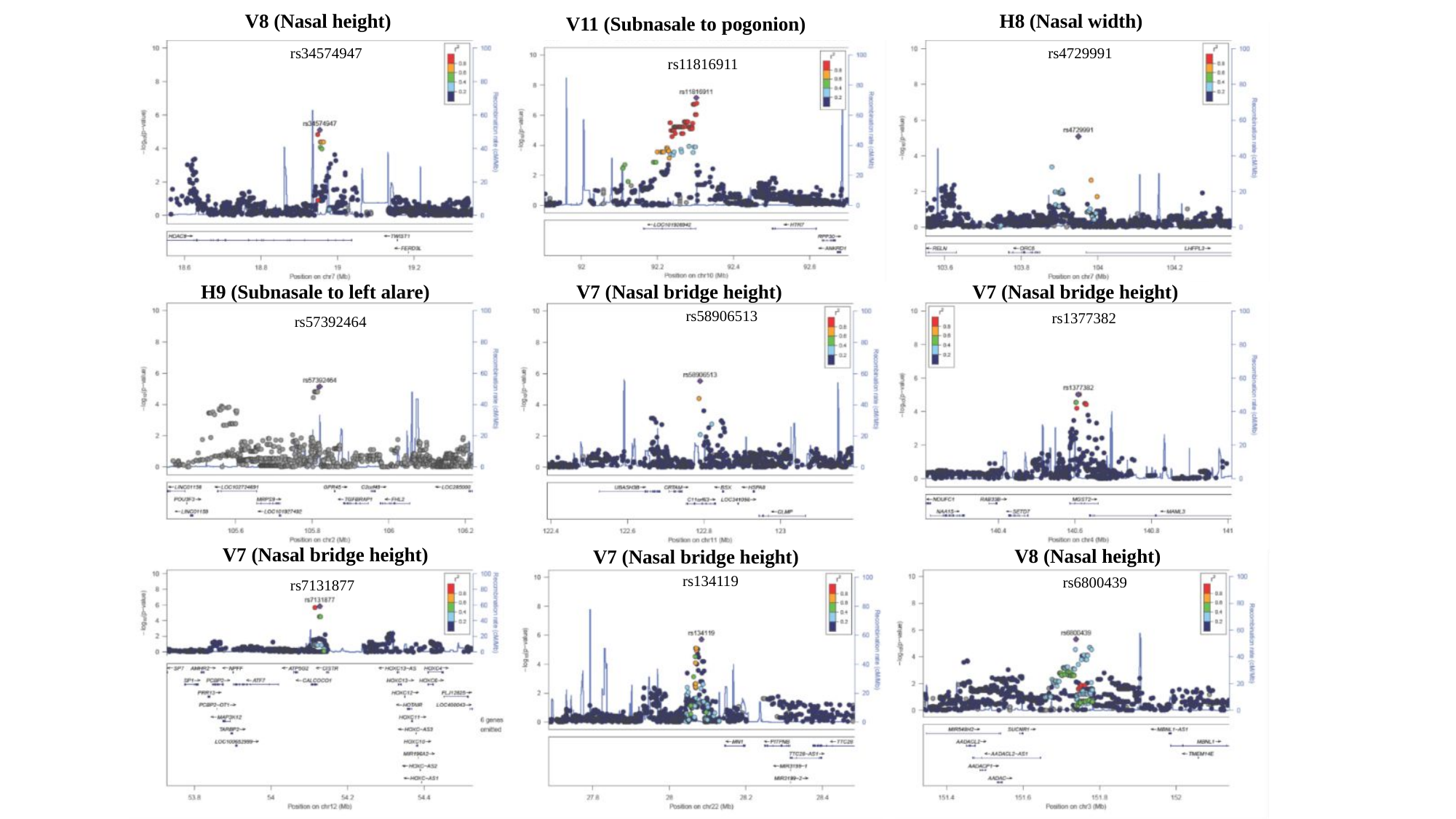

V8 (Nasal height)
H8 (Nasal width)
V11 (Subnasale to pogonion)
rs34574947
rs4729991
rs11816911
V7 (Nasal bridge height)
V7 (Nasal bridge height)
H9 (Subnasale to left alare)
rs58906513
rs1377382
rs57392464
V7 (Nasal bridge height)
V8 (Nasal height)
V7 (Nasal bridge height)
rs134119
rs6800439
rs7131877

## Slide 8
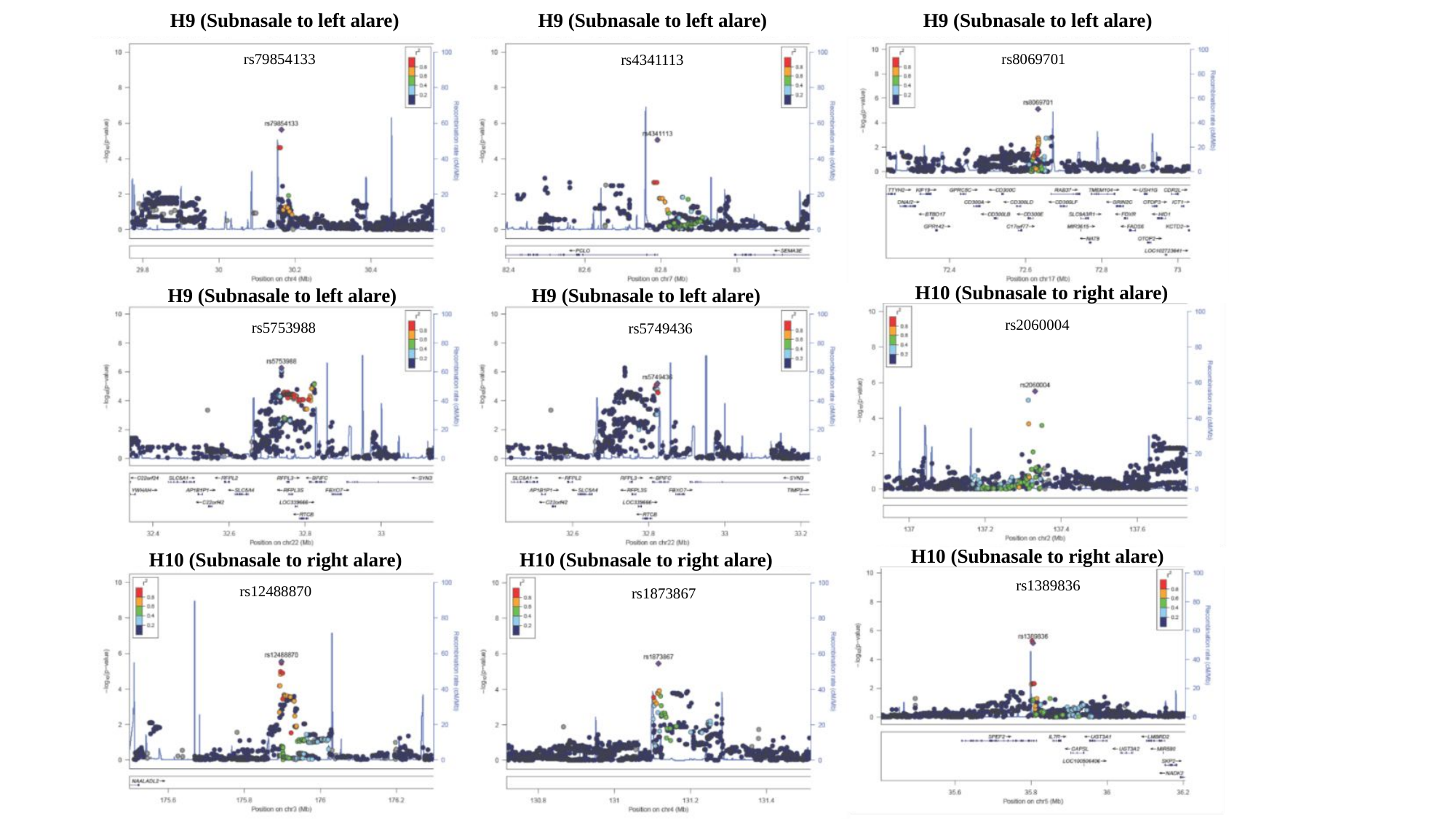

H9 (Subnasale to left alare)
H9 (Subnasale to left alare)
H9 (Subnasale to left alare)
rs79854133
rs8069701
rs4341113
H10 (Subnasale to right alare)
H9 (Subnasale to left alare)
H9 (Subnasale to left alare)
rs2060004
rs5753988
rs5749436
H10 (Subnasale to right alare)
H10 (Subnasale to right alare)
H10 (Subnasale to right alare)
rs1389836
rs12488870
rs1873867

## Slide 9
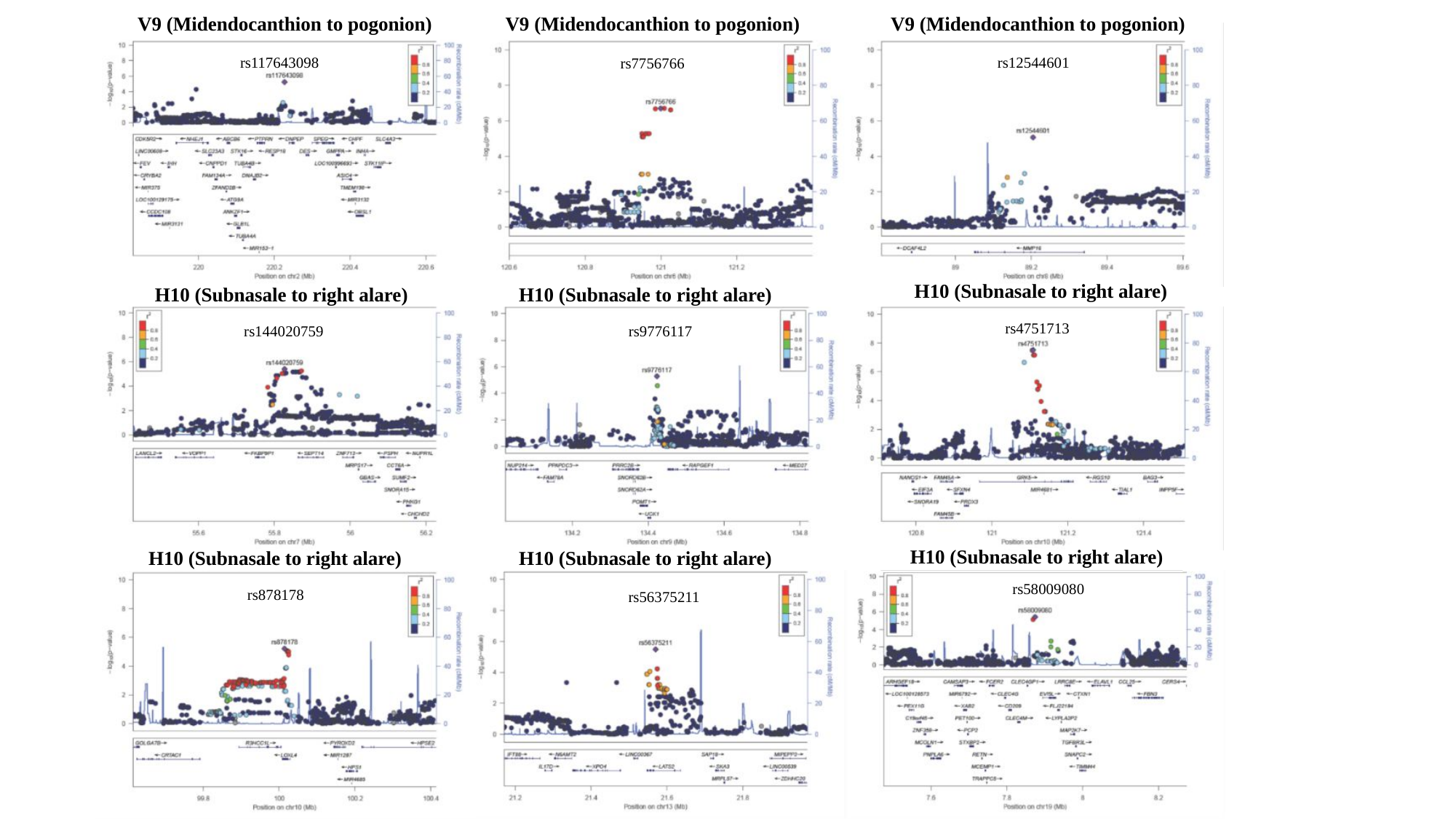

V9 (Midendocanthion to pogonion)
V9 (Midendocanthion to pogonion)
V9 (Midendocanthion to pogonion)
rs117643098
rs12544601
rs7756766
H10 (Subnasale to right alare)
H10 (Subnasale to right alare)
H10 (Subnasale to right alare)
rs4751713
rs144020759
rs9776117
H10 (Subnasale to right alare)
H10 (Subnasale to right alare)
H10 (Subnasale to right alare)
rs58009080
rs878178
rs56375211

## Slide 10
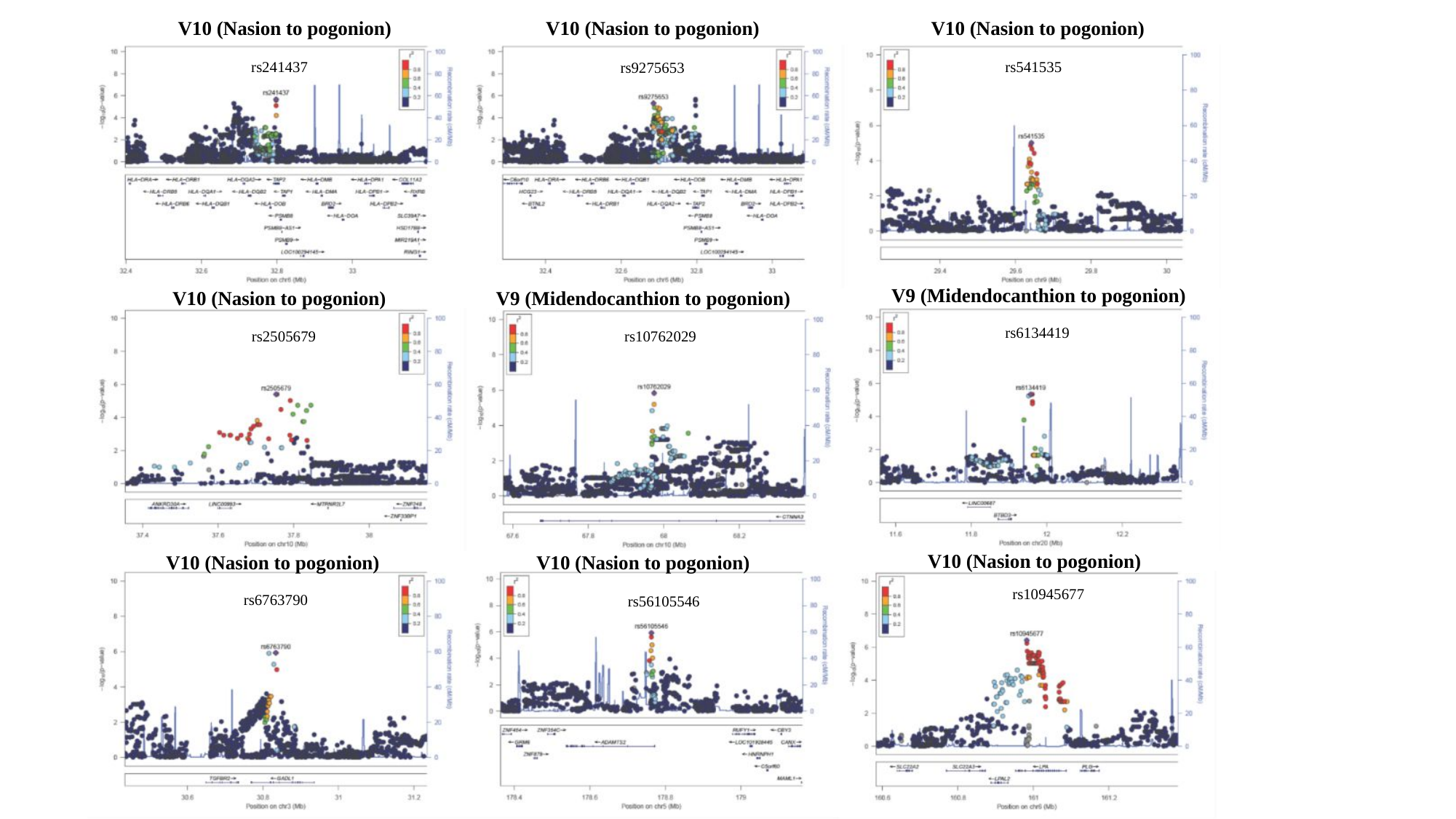

V10 (Nasion to pogonion)
V10 (Nasion to pogonion)
V10 (Nasion to pogonion)
rs241437
rs541535
rs9275653
V9 (Midendocanthion to pogonion)
V9 (Midendocanthion to pogonion)
V10 (Nasion to pogonion)
rs6134419
rs2505679
rs10762029
V10 (Nasion to pogonion)
V10 (Nasion to pogonion)
V10 (Nasion to pogonion)
rs10945677
rs6763790
rs56105546

## Slide 11
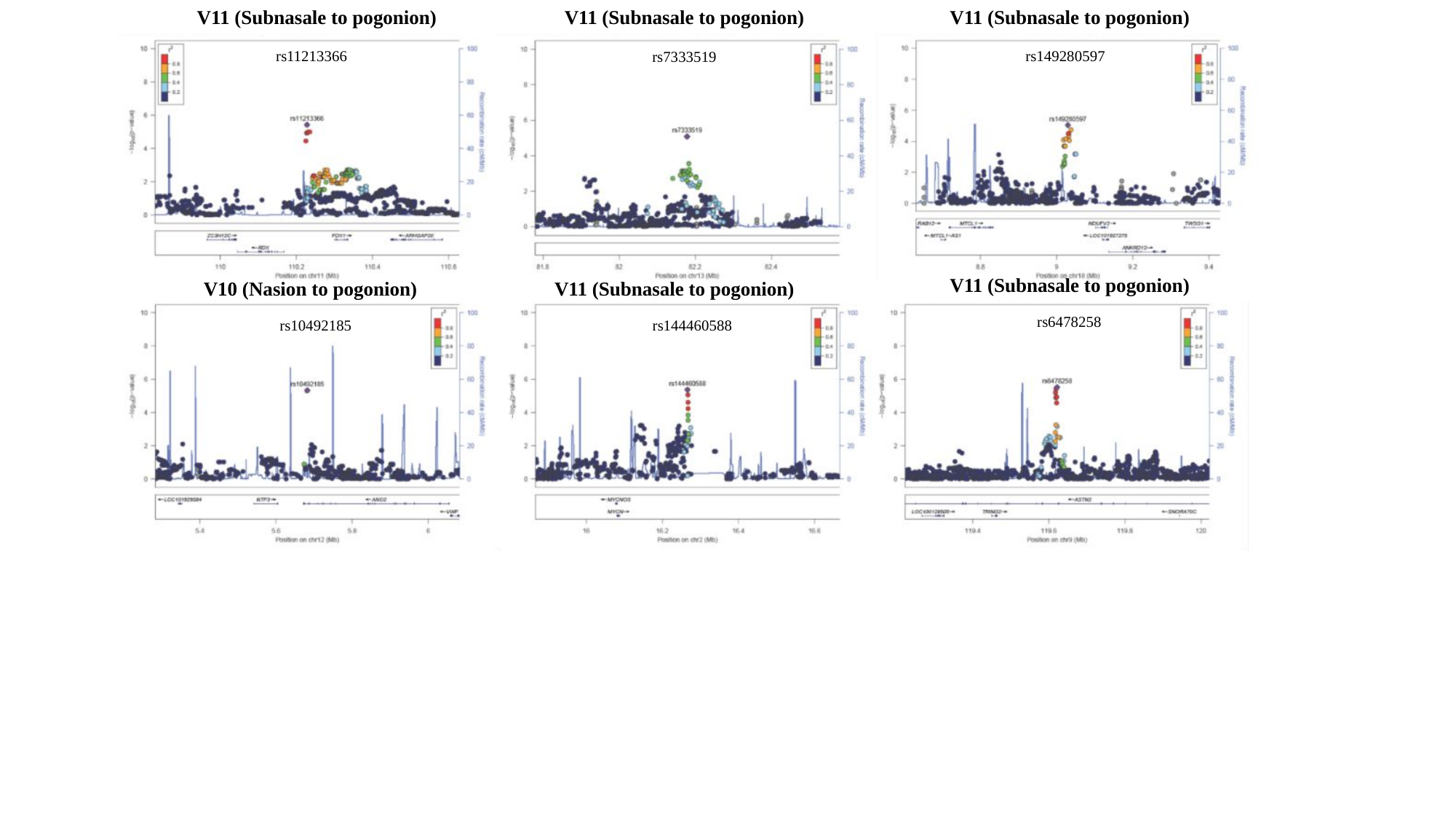

V11 (Subnasale to pogonion)
V11 (Subnasale to pogonion)
V11 (Subnasale to pogonion)
rs11213366
rs149280597
rs7333519
V11 (Subnasale to pogonion)
V11 (Subnasale to pogonion)
V10 (Nasion to pogonion)
rs6478258
rs10492185
rs144460588
