# Supplementary figures and images for: Classification of early age facial growth pattern and identification of the genetic basis in two Korean populations
Source: Sci Rep. 2022 Aug 15;12:13828. doi: 10.1038/s41598-022-18127-6 (PMC9378761; doi:10.1038/s41598-022-18127-6)

**Figure S2. QQ plot of the combined GWAS analysis for 21 facial phenotypes.**

**
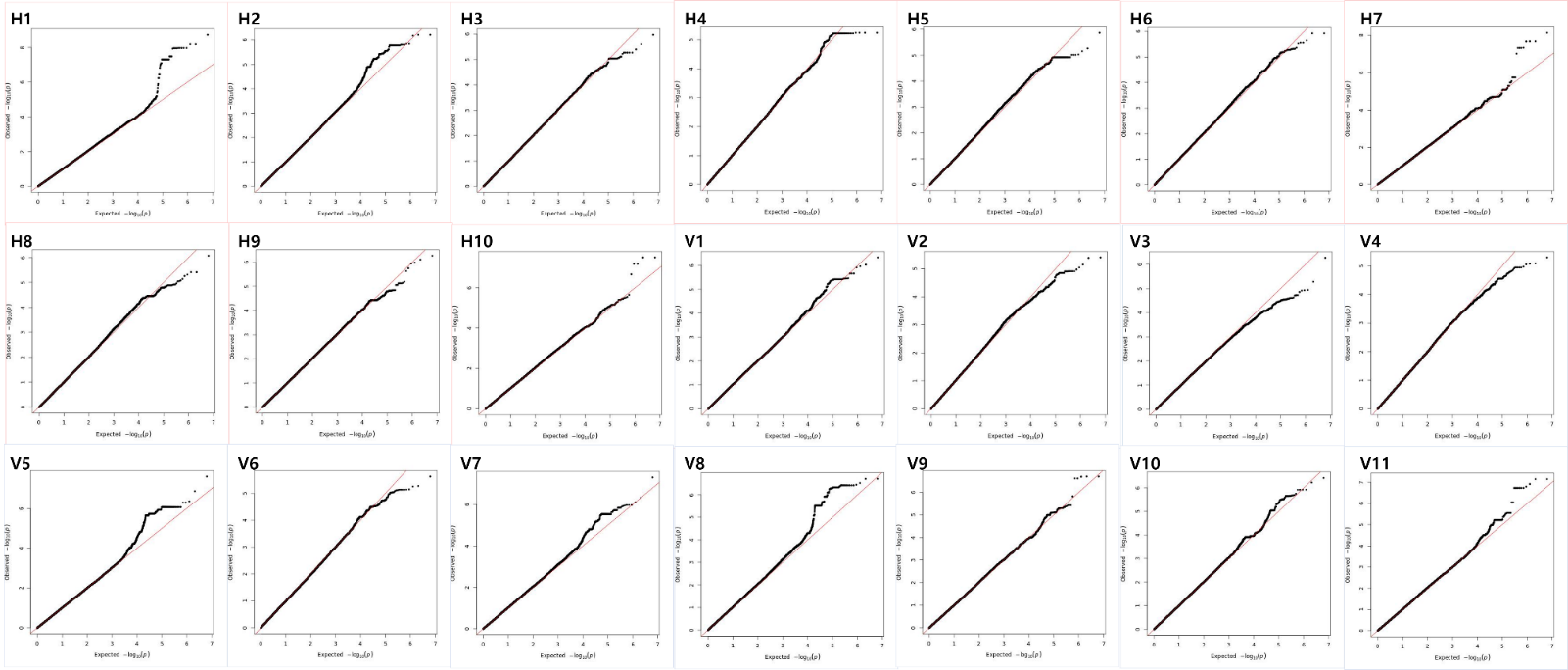
**

Supplement: Supplementary file 1 — Supplementary Information. [file 41598_2022_18127_MOESM1_ESM.zip › Supplementary Figure 2.docx]
